# Functional investigation of Zur in metal ion homeostasis, motility and multiple stresses resistance in cyanobacteria *Synechocystis* sp. PCC 6803

**DOI:** 10.1007/s44154-025-00224-x

**Published:** 2025-05-07

**Authors:** Han Jin, Xiaoru Han, Chen Zheng, Jingling Xu, Wenjing Zhang, Yanchao Gu, Ying Peng, Jiaxin Han, Lei Xu, Xihui Shen, Yantao Yang

**Affiliations:** State Key Laboratory for Crop Stress Resistance and High-Efficiency Production, Shaanxi Key Laboratory of Agricultural and Environmental Microbiology, College of Life Sciences, Northwest A&F University, Yangling, 712100 China

**Keywords:** Zur, Transcriptional regulator, Metal ion homeostasis, Motility, Stress resistance, *Synechocystis* sp. PCC 6803

## Abstract

Zur (zinc uptake regulator), a member of the Fur (ferric uptake regulator) family of transcriptional regulators, plays multifaceted roles by regulating the gene expressions, such as modulating zinc ion uptake by regulating the *znuABC* gene cluster and influencing bacterial motility by modulating genes associated with flagella or pili. The photosynthetic autotroph *Synechocystis* sp. PCC 6803 is frequently used as an indicator organism for water pollution and a cell factory for high-value biochemical production in synthetic biology. During its growth, this organism often encounters various abiotic stresses, including oxidative, salt, and antibiotic stress. In this study, we conducted transcriptomic analysis on both Δ*zur* mutant and wild-type (WT) strains to identify potential Zur-regulated genes in *Synechocystis* sp. PCC 6803. These genes primarily participate in multiple pathways such as inorganic ion transport, carbohydrate transport, energy production and conversion, and cell motility. Zur not only controls zinc ion homeostasis within the cell but also influences the iron balance by directly regulating the expression of the *fur* gene. In terms of motility, Zur regulates the expression of bacterial pili gene cluster and other motility-related genes, thereby affecting the twitching motility of *Synechocystis* sp. PCC 6803. Furthermore, Zur plays a crucial role in promoting biofilm formation and enhancing resistance to salt, oxidative, and antibiotic stresses by modulating relative gene expression. In conclusion, as a global transcriptional regulator, Zur plays pivotal roles in metal ion homeostasis, motility, and resistance to multiple stresses in *Synechocystis* sp. PCC 6803. This study illustrates the Zur regulons in *Synechocystis* sp. PCC 6803, and underscores the importance of Zur in enhancing the environmental adaptability of cyanobacteria.

**Supplementary Information:**

The online version contains supplementary material available at 10.1007/s44154-025-00224-x.

## Highlights

1. Transcriptome analysis of Zur’s regulons in *Synechocystis* sp. PCC 6803.

2. Zur mediates metal homeostasis beyond zinc.

3. Zur modulates pili biosynthesis and subsequent motility.

4. Zur promotes biofilm formation and stresses resistance.

**Supplementary Information:**

The online version contains supplementary material available at 10.1007/s44154-025-00224-x.

## Introduction

Bacteria encounter substantial challenges in their survival within the natural environment due to external environmental stresses, such as temperature fluctuations, osmotic pressure variations, and threats from metal ions (Kristensen et al. 2020). The role of transcriptional regulators is crucial in enabling these organisms to adapt to these changes, thereby ensuring their survival (Browning and Busby 2016). Metal ions play a key role in bacterial growth as they function as enzyme cofactors involved in most biochemical reactions. However, an excess of metals can be detrimental to cells, underscoring the importance of maintaining homeostasis of metal ions in vivo. The Fur family of transcriptional regulators is instrumental in modulating the uptake, storage, and efflux of metal ions, thereby controlling their metabolism (Helmann 2014). These regulators also significantly influence resistance to acidic and oxidative stress, and are implicated in the regulation of virulence factors, all of which are essential for bacterial survival and adaptation (Askoura et al. 2016; Troxell and Hassan 2013). Within the Fur family, there are numerous transcriptional regulators with diverse biological functions. For instance, Fur acts as a global regulator, managing iron homeostasis and responding to various stresses. Similarly, Zur, Nur, and Mur can regulate Zn^2+^, Ni^2+^ and Mn^2+^, respectively. Additionally, PerR and Irr can sense peroxide and haem respectively (Sevilla et al. 2021; Ahn et al. 2006; Díaz-Mireles et al. 2004; Jacquamet et al. 2009; Hamza et al. 1998).

Within the Fur family, both Fur and Zur have been extensively studied and reported. Furs are Fe^2+^-dependent DNA-binding proteins that are commonly associated with iron limitation, oxidative stress, bacterial virulence, and pathogenicity (Pinochet-Barros and Helmann 2018; Troxell et al. 2013; Zuo et al. 2023). Fur proteins typically consist of an N-terminal DNA-binding domain that binds to metal ions or to specific DNA, and a C-terminal dimerisation domain that stabilises their dimeric or tetrameric structure. These two domains are linked by a flexible hinge region that regulates conformation (Sevilla et al. 2021; Fillat 2014). This structure enables Fur to form dimers with Fe^2+^, which in turn regulates the expression of specific genes by binding the Fur-boxes in the promoter region of target genes (Fillat 2014; Shin et al. 2007). The Zur protein shares structural and functional similarities with Fur. It contains two or three zinc-binding sites and a helix-turn-helix (HTH) motif in its N-terminal, which are capable of sensing zinc ions and interacting with the major and minor grooves of the DNA (Ghassemian and Straus 1996). Zur plays a pivotal role in the regulation of Zn^2+^ uptake and efflux. At elevated concentrations of Zn^2+^ in vivo, Zur inhibits the expression of the Zn^2+^ uptake genes such as *znuABC* and activates the Zn^2+^ efflux gene such as *zitB* by binding to their respective promoters (Kandari et al. 2021). Conversely, these processes are reversed at low Zn^2+^ concentrations to maintain Zn^2+^ homeostasis (Choi et al. 2017). In *Streptomyces coelicolor*, Zur maintains intracellular zinc homeostasis by inhibiting the expression of zinc import genes (*znuABC*) and promoting the expression of the zinc export gene (*zitB*) in response to elevated zinc ion concentrations (Choi et al. 2017). In *Pseudomonas aeruginosa*, Zur binds to precise DNA sequences known as Zur box, repressing the expression of genes (*znuABC*) responsible for zinc import, and activating the transcription of *czcR*, which promotes the expression of zinc export genes such as *czcCBA* and *cadA* (Ducret et al. 2023). Similarly, in *Caulobacter crescentus*, Zur directly binds to the promoter regions of zinc uptake genes (*znuGHI*, *znuK*, *znuL*, *znuM*, and *zrpW*), repressing their expression in the presence of zinc to reduce its uptake. Concurrently, Zur activates the expression of zinc efflux genes (*czrCBA* and *zntA*), promoting zinc expulsion and maintaining intracellular zinc homeostasis (Mazzon et al. 2014). Furthermore, Zur is also involved in regulating the balance of iron and other metal ions in vivo, affecting cell motility, virulence factors, oxidative resistance, antibiotic resistance, and adaptation to temperature changes (Patzer and Hantke 1998; Rodionov et al. 2004; Campoy et al. 2002; Schröder et al. 2010; Haas et al. 2009; Ajiboye et al. 2019). The diverse functions of Zur contribute to bacterial survival in extreme environments, making the study of Zur proteins in bacteria of significant interest.

Cyanobacteria, the sole group of prokaryotic organisms on Earth capable of aerobic photosynthesis, utilize sunlight, water, and carbon dioxide as substrates to generate organic substances that store chemical energy and release oxygen. They serve as the main primary producers in aquatic food chains and play an important role in biogeochemical cycles. Among them, *Synechocystis* sp. PCC 6803, a unicellular cyanobacterium equipped with a natural DNA transformation system, is one of the most extensively used model organisms due to its manipulability. Currently, the Zur protein of *Anabaena* sp. PCC 7120 has been extensively studied in cyanobacteria. Zur modulates zinc homeostasis in *Anabaena* sp. strain PCC 7120 by adjusting zinc-DNA binding in response to environmental changes in cyanobacteria (Sein-Echaluce et al. 2018; Napolitano et al. 2012). It also modulates the expression of antioxidant enzyme genes to enhance the antioxidant capacity in *Anabaena* sp. strain PCC 7120 (Sein-Echaluce et al. 2015; López-Gomollón et al. 2009). Transcriptome sequencing of the Δ*zur* mutant and the parent strains revealed that Zur regulates enzymes associated with the synthesis and transport of envelope polysaccharide layer, which affects the heterocyst development and biofilm formation in *Anabaena* sp. strain PCC 7120(Olivan-Muro et al. 2023). It has been mentioned that Zur, encoded by *sll1937*, binds to the promoter of the *znuABC* gene cluster in *Synechocystis* sp. PCC 6803 (Tottey et al. 2012). However, it remains unclear how Zur regulates zinc transport and whether it has additional functions. Therefore, a comprehensive investigation into the precise functions of Zur in *Synechocystis* sp. PCC 6803 is urgently required.

In this study, RNA-seq was conducted on both the WT and Δ*zur* mutant strains of *Synechocystis* sp. PCC 6803 to identify potential Zur-regulated genes. The transcriptome sequencing results were subsequently validated using qRT-PCR and electrophoretic mobility shift assay (EMSA). A range of stress treatments were applied to the WT, Δ*zur* mutant, and Zur overexpression strains in order to investigate the roles of Zur in stress resistance. The findings indicated that Zur plays a crucial role in various physiological processes, including ion transportation, oxidative, osmatic, and antibiotic stress resistances, biofilm formation, and motility in *Synechocystis* sp. PCC 6803. In conclusion, these findings suggest that Zur can enhance the environmental adaptation of *Synechocystis* sp. PCC 6803.

## Results

### Genome-wide analysis of the genes regulated by Zur

**Fig. 1:**
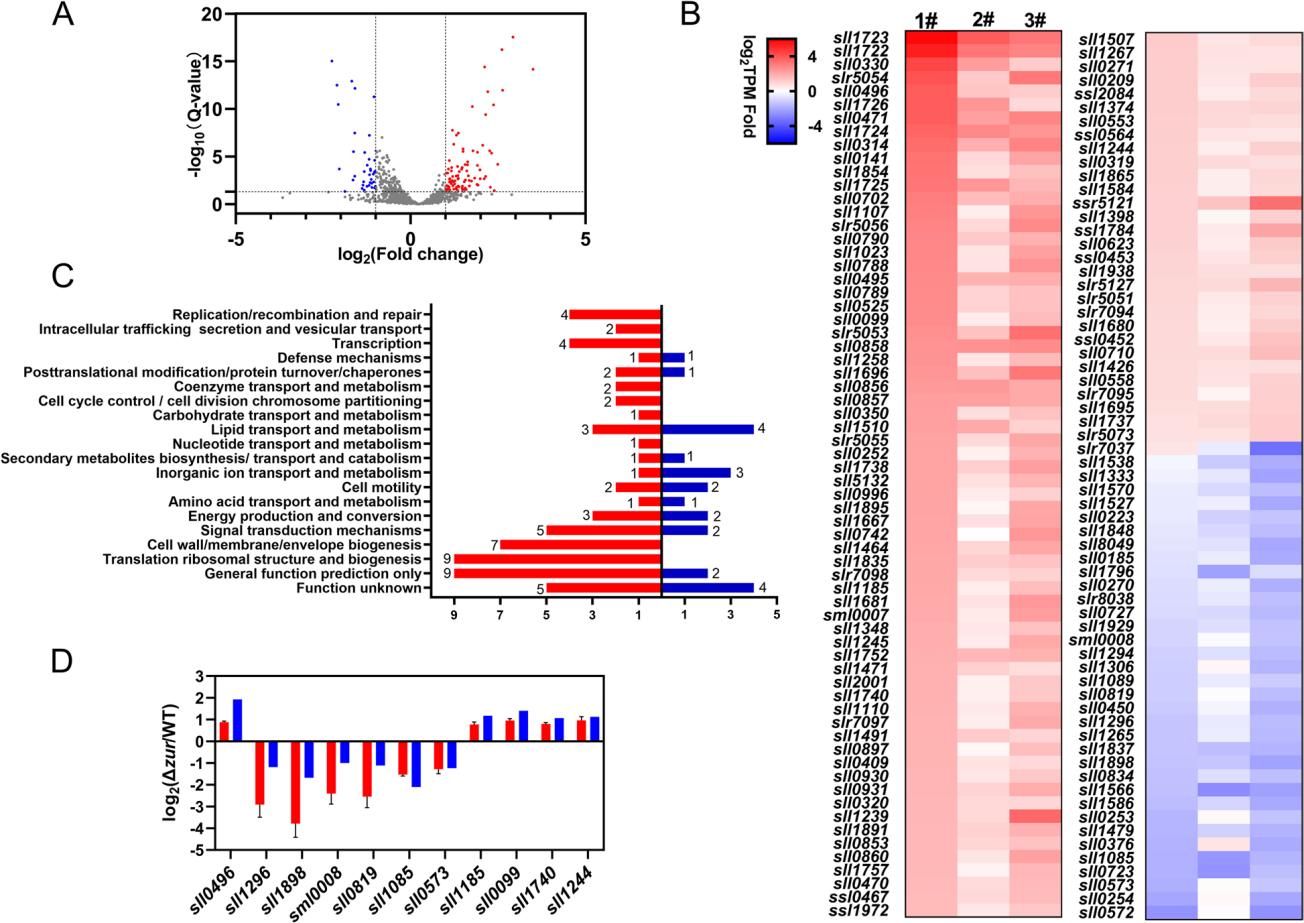
Genome-wide analysis of the genes regulated by Zur. **A** Volcano plot depicting gene expression analysis WT or Δ*zur* mutant strain. The x-axis represents the fold change in gene expression, calculated as log_2_(Δ*zur* TPM/WT TPM), and the y-axis signifies the statistical significance, represented by -log_10_(Q-value). Broken lines indicate a Q-value threshold of 0.05 and a log_2_(fold change) threshold of 1 and -1. Downregulated genes are represented in blue, upregulated genes in red, and genes with no significant difference in expression are depicted in grey. **B** A heatmap displays DEGs according to KEGG annotation. Downregulated genes are represented in blue, upregulated genes in red with gene expression calculated as log_2_(Δ*zur* TPM/WT TPM). **C** COG pathway enrichment analysis of DEGs, with upregulated genes by Zur depicted in red and downregulated genes in blue. **D** qRT-PCR validation of RNA-Seq data. Eleven genes were selected for verifying the RNA-seq data through qRT-PCR. The red bars represent qRT-PCR data, while the blue bars represent RNA-seq data. Each group includes three biological replicates

Zur transcriptional regulators have been previously reported to possess a variety of biological functions, including the maintenance of metal ion homeostasis, influence on biofilm formation, promotion of motility, and enhancement of resistance to oxygen stress (Gu et al. 2024a). The protein encoded by *sll1937* was identified as Zur in *Synechocystis* sp. PCC 6803 (Barnett et al. 2012; Olivan-Muro et al. 2023). To investigate the global regulatory function of Zur in *Synechocystis* sp. PCC 6803, RNA was extracted from both WT and Δ*zur* mutant strains during their exponential phase, followed by RNA-seq analysis (SRA accession: PRJNA1174049). The RNA-seq data was processed, and the differentially expressed genes (DEGs) were screened at a Q-value < 0.05 and |log_2_ (fold change)|> 1. A total of 141 DEGs (Table S2), comprising 102 upregulated and 39 downregulated, were identified in the Δ*zur* mutant strain (Fig. 1A). A heatmap plotting the 131 DEGs annotated by the Kyoto Encyclopedia of Genes and Genomes (KEGG) is presented in Fig. 1B, while the Cluster of Orthologous Groups of proteins (COG) pathway enrichment analysis result is illustrated in Fig. 1C. The DEGs were clustered into 20 pathways as per the COG enrichment pathway analysis, which included inorganic ion transport and metabolism, carbohydrate transport and metabolism, energy production and conversion, cell motility, among others. To validate the RNA-seq results, the expression of representative downregulated *sll1296* (hybrid sensor histidine kinase/response regulator)*, sll1898* (heme A synthase)*, sml0008* (photosystem I reaction center subunit IX)*, sll0819* (photosystem I reaction center subunit III)*, sll1085* (glycerol-3-phosphate dehydrogenase)*, sll0573* (carbamate kinase) and upregulated *sll0496* (LptF/LptG family permease)*, sll1185* (oxygen-dependent coproporphyrinogen oxidase)*, sll0099* (precorrin-6B C5,15-methyltransferase)*, sll1740* (50S ribosomal protein L19)*, sll1244* (50S ribosomal protein L9) genes were confirmed by qRT-PCR (Fig. 1D), thereby suggesting that the RNA-seq analysis is reliable. In conclusion, Zur functions as a global transcriptional regulator in multiple pathways in *Synechocystis* sp. PCC 6803.

### Zur orchestrates the homeostasis of Zinc and iron in vivo

**Fig. 2:**
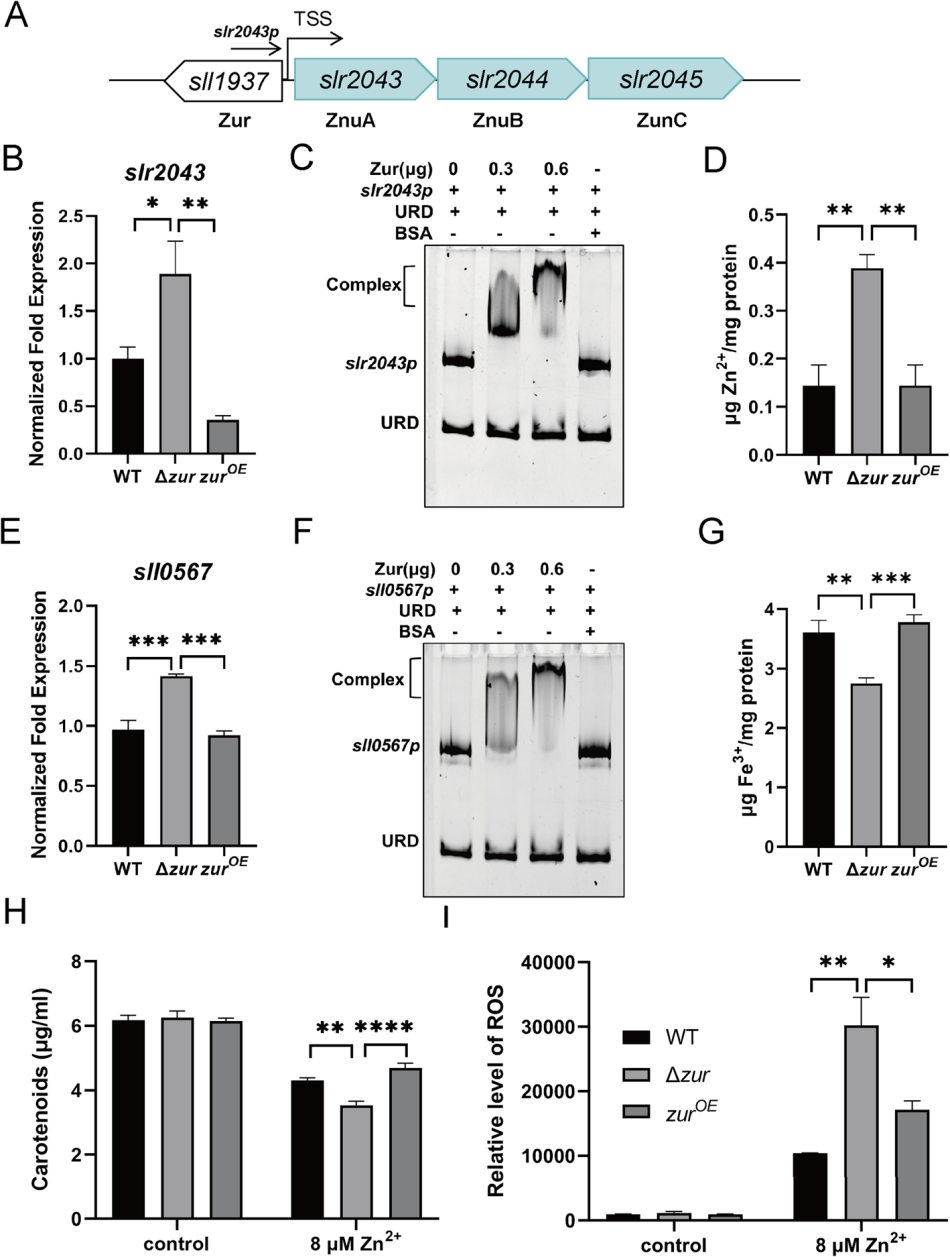
Zur can regulate Zn^2+^ transportation. **A** The schematic illustrates the organization of the *znuABC* gene cluster, with the predicted TSS within the gene cluster annotated and arrows indicating the direction of transcription. **B** qRT-PCR analysis was conducted to determine the relative expression levels of *slr2043*. **C** EMSA demonstrated the binding of His6-Zur to the *slr2043* promoter, with varying Zur concentrations (0, 0.3, 0.6 μg), 30 ng promoter DNA fragments, or unrelated-DNA fragment (URD) in each lane. **D** The intracellular concentration of Zn^2+^ in the WT, Δ*zur* mutant and Zur overexpression strains of *Synechocystis* sp. PCC 6803 under conditions of 8 μM Zn^2+^ stress was measured. **E** qRT-PCR analysis was used to ascertain the relative expression levels of *sll0567*. **F** EMSA revealed that His6-Zur binds to the *sll0567* promoter, with different Zur concentrations (0, 0.3, 0.6 μg), 30 ng promoter DNA fragments, or URD in each lane. **G** The intracellular concentration of Fe^3+^ in the WT, Δ*zur* mutant, and overexpression strains of *Synechocystis* sp. PCC 6803 under conditions of 8 μM Zn^2+^ stress was measured. **H** The content of carotenoids in the WT, Δ*zur* mutant, and Zur overexpression strains grown in BG11 medium, with or without 8 μM Zn^2+^. The black bars indicate the WT strain, the light grey bars indicate the Δ*zur* mutant strain, and the dark grey bars indicate the Zur overexpression strain. **I** The relative levels of ROS in the WT, Δ*zur* mutant and Zur overexpression strains under control and 8 μM Zn^2+^ stress conditions. Error bars represent ± SEM (*n* = 3). *P < 0.05; **P < 0.01; ***P < 0.001; ****P < 0.0001

Previous research has demonstrated that Zur negatively regulates the zinc uptake transportor gene cluster, *znuABC*, to maintain zinc homeostasis in vivo (Patzer and Hantke 1998). The *zur* (*sll1937*) gene is situated within the *znuABC* (*slr2043-2045*) gene cluster in *Synechocystis* sp. PCC 6803 (Fig. 2A). Given that the promoter region of the *znuABC* gene is located upstream of the *sll1937* gene, we constructed a Δ*zur*^91−138^ mutant strain, which retains the promoter region of *znuABC*, to investigate the regulatory role of Zur on metal ions. The zinc concentrations that induce stress in *Synechocystis* sp. PCC6803 strains have been determined to be 8 μM (Fig.S4). RNA was extracted from the WT, Δ*zur* mutant, and Zur overexpression strains grown in BG11 medium with 8 μM Zn^2+^, and qRT-PCR was performed to analyze the relative expression levels of *znuA* (*slr2043*) gene. As anticipated, the expression of *znuA* in Δ*zur* mutant was significantly higher than in the WT and Zur overexpression strains (Fig. 2B). We confirmed that Zur negatively regulates the expression of the *znuA* gene by directly binding to its promoter through EMSA (Fig. 2C and Fig. S2A). Accordingly, the intracellular Zn^2+^ concentration in the Δ*zur* mutant was notably higher than that in the WT and Zur overexpression strains under 8 μM Zn^2+^ stress (Fig. 2D). A putative cross-talk between the Zur and Fur regulatory networks has been reported in bacteria (Mazzon et al. 2014). In the transcriptomic results, the expression of the *fur* (*sll0576*) gene was regulated by Zur with a log_2_(Δ*zur*/WT) fold change of 0.77. It was demonstrated that Zur can negatively regulate the expression of the *fur* gene by directly binding to its promoter through qRT-PCR and EMSA (Fig. 2E, F and Fig. S2B). The intracellular Fe^3+^ content in the Δ*zur* mutant was significantly lower than that in the WT and Zur overexpression strains (Fig. 2G). These findings suggest that Zur can orchestrate ferric balance by regulating the ferric uptake regulator Fur. To assess the impact of Zur on the growth of *Synechocystis* sp. PCC 6803 under 8 μM Zn^2+^ stress, we measured the levels of photosynthetic pigments and reactive oxygen species (ROS) in the WT, Δ*zur* mutant, and Zur overexpression strains. The results showed a significant reduction in carotenoid content and a notable elevation in ROS levels in the Δ*zur* mutant compared to the WT and Zur overexpression strains following stress exposure (Fig. 2H and I).

### Zur promotes motility by upregulating the expression of motility related genes

**Fig. 3:**
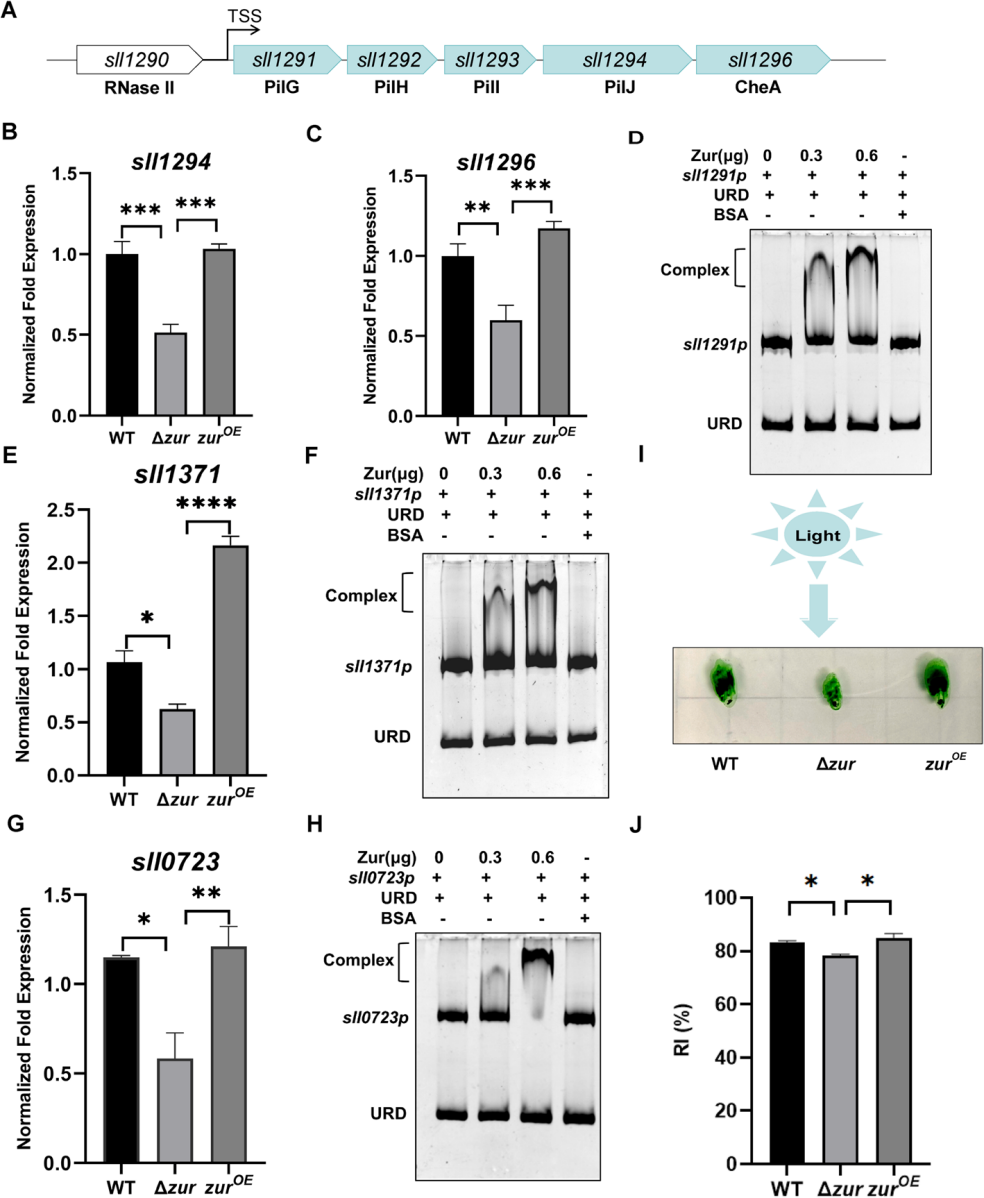
Zur promotes the motility of *Synechocystis* sp. PCC 6803. **A** The schematic illustrates the organization of the *sll1291* gene cluster, with the predicted TSS within the gene cluster annotated and arrows indicating the direction of transcription. **B** and **C** qRT-PCR analyzes the relative expression levels of *sll1294* and *sll1296*. **D** EMSA analysis indicates His6-Zur binding to the promoter of *sll1291* gene cluster, with varying Zur concentrations (0, 0.3, 0.6 μg), 30 ng DNA fragments, and URD in each lane. **E** qRT-PCR reveals the relative expression levels of *sll1371*. **F** EMSA analysis shows His6-Zur binding to the *sll1371* promoter, with different Zur concentrations (0, 0.3, 0.6 μg), 30 ng DNA fragments, and URD in each lane. **G** qRT-PCR represents the relative expression levels of *sll0723*. **H** EMSA analysis reveals His6-Zur binding to the *sll0723* promoter, with varying Zur concentrations (0, 0.3, 0.6 μg), 30 ng DNA fragments, and URD in each lane. **I** Phototactic motility assays of *Synechocystis* sp. PCC 6803. Cultures of WT, Δ*zur* mutant and Zur overexpression strains were diluted with fresh BG11 medium to an OD_730_ of 0.6 and spotted onto twitching plates for 10 days. The blue arrow denotes the direction of the light source. **J** The twitching motility distance from the inoculation point to the colony edges nearest (D1) and furthest (D2) from the light source was measured, and the response index (RI) was calculated using the formula: RI = D1 / (D1 + D2). Error bars represent ± SEM (*n* = 3). *P < 0.05; **P < 0.01; ***P < 0.001; ****P < 0.0001

In previous studies, Zur has been reported to regulate bacterial motility (Chen et al. 2023a). The gene*s pilGHI,* which influence the biosynthesis of Type IV pili (T4P), play a role in the regulation of bacterial twitching motility (Zhou et al. 2015; Corral et al. 2020). In our RNA-seq analysis, we observed that Zur can upregulate the expression of genes associated with the T4P (Fig. 3A). qRT-PCR was conducted to validate the transcriptional regulation by Zur of *sll1294* and *sll1296*, which encode PilG and CheA, respectively (Fig. 3B and C). Additionally, we also have demonstrated that Zur directly interacts with the promoter region of the *sll1291* gene cluster as confirmed using EMSA (Fig. 3D and Fig. S2C). Though the gene cluster of *sll1291*-*96* may not be the predominant one functioning in motility (Bhaya et al. 2001), it has been definitively shown to be directly regulated by Zur. Furthermore, Zur also regulates the expression of the *sll1371* and *sll0723* genes, with log_2_(Δ*zur*/WT) fold changes of -0.43 and -2.07, respectively. The *sll1371* gene encodes Sycrp1, a cAMP receptor protein implicated in bacterial motility (Song et al. 2018). The protein encoded by *sll0723* gene contains the DUF4114 domain, which can be activated by binding Ca^2+^, thereby regulating bacterial motility (Xue et al. 2022). We employed qRT-PCR and EMSA to validate the direct regulation of these DEGs (Fig. 3E, F, G, H and Fig. S2D, E). Motility assays were performed on the WT, Δ*zur* mutant, and Zur-overexpression strains to elucidate the role of Zur in bacterial twitching motility. The results showed that the twitching motility capability of the mutant strain was significantly diminished compared to both WT and overexpression strains (Fig. 3I and J). Zur enhances the motility of *Synechocystis* sp. PCC 6803 by directly modulating the motility-related genes.

### Zur enhances resistance to salt stress by upregulating the expression of GGPS

**Fig. 4:**
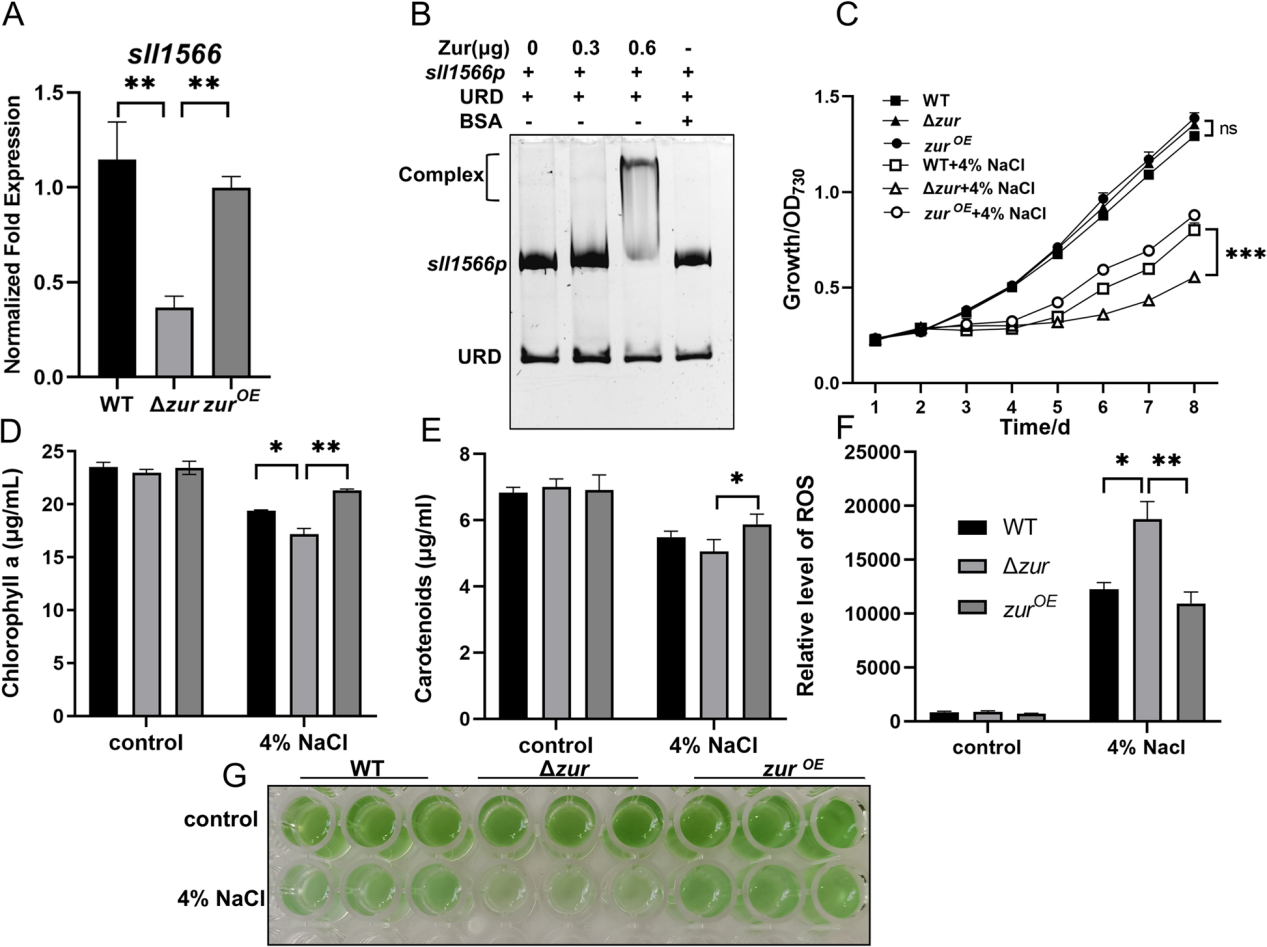
Zur enhances resistance to salt stress. **A** qRT-PCR analysis of the relative expression levels of *sll1566*. **B** EMSA analysis reveals the binding of His6-Zur to the *sll1566* promoter, with varying Zur concentrations (0, 0.3, 0.6 μg), 30 ng DNA fragments, and URD in each lane. **C** Growth curves for the WT, Δ*zur* mutant and Zur overexpression strains in media with or without 4% NaCl. The x-axis indicates time and y-axis indicates OD_730_. **D** and **E** The levels of chlorophyll a and carotenoids in the WT, Δ*zur* mutant and Zur overexpression strains under control and 4% NaCl stress conditions. **F** The relative levels of ROS in the WT, Δ*zur* mutant and Zur overexpression strains under control and 4% NaCl stress conditions. **G** Cultures of the WT, Δ*zur* mutant and Zur overexpression strains under control and 4% NaCl stress conditions. Error bars represent ± SEM (*n* = 3). *P < 0.05; **P < 0.01; ***P < 0.001; ns, not significant

The *sll1566* gene encodes glucosylglycerol-phosphate synthase (GGPS), a key enzyme involved in osmolyte synthesis that can resist salt stress (Marin et al. 1998). Zur can upregulate the expression of *sll1566,* as evidenced by a log_2_(Δ*zur*/WT) foldchange of -2.25 in RNA-seq analysis**.** qRT-PCR was conducted to validate the expression of *sll1566* in the WT, Δ*zur,* and Zur overexpression strains (Fig. 4A). The expression was suppressed in Δ*zur* and restored in the Zur overexpression strain. The interaction of Zur with the promoter region of *sll1566* was confirmed by EMSA (Fig. 4B and Fig. S2F), suggesting that Zur regulates the expression of *sll1566* by directly binding to its promoter region. To investigate the role of Zur in salt stress resistance, the WT, Δ*zur* mutant, and Zur overexpression strains were cultured in liquid BG11 medium or BG11 supplemented with 4% NaCl. No significant growth difference was observed among these three strains in the control BG11 medium, whereas the Δ*zur* mutant strain exhibited a significant decrease in growth rate under the salt stress conditions compared to both the WT and overexpression strains (Fig. 4C, G and Fig. S3A). After an eight-day cultivation period, the photosynthetic pigment contents and the ROS levels in the WT, Δ*zur* mutant, and Zur overexpression strains were quantified. The results indicated that the photosynthetic pigment contents and ROS levels were essentially equivalent among WT, Δ*zur* mutant, and Zur overexpression strains in control BG11 medium. However, upon exposure to 4% NaCl stress, the contents of chlorophyll a and carotenoids in the Zur overexpression strain were significantly higher than those in the Δ*zur* mutant strain (Fig. 4D and E). Concurrently, the levels of ROS were significantly lower in WT and Zur overexpression strains than in the Δ*zur* mutant strain (Fig. 4F). These results suggest that Zur improves salt stress resistance by upregulating the expression of *sll1566* gene, which in turn promotes glucosylglycerol synthesis in *Synechocystis* sp. PCC 6803.

### Zur facilitates the resistance to oxidative stress in *Synechocystis* sp. PCC 6803

**Fig. 5:**
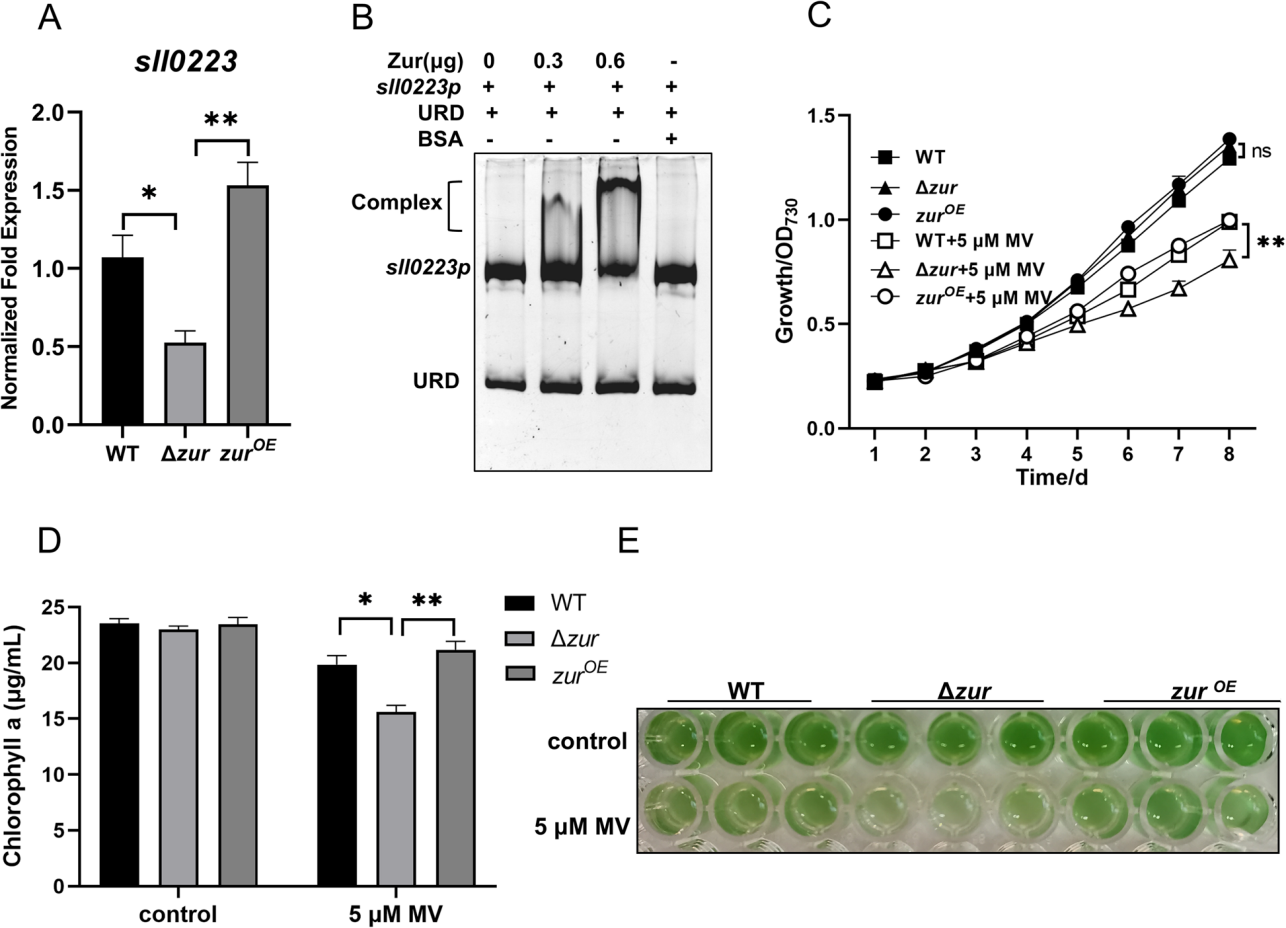
Zur facilitates the resistance to oxidative stress. **A** qRT-PCR analysis of the relative expression levels of *sll0223*. **B** EMSA analyzed the binding of His6-Zur to the *sll0223* promoter, with different Zur concentrations (0, 0.3, 0.6 μg), 30 ng DNA fragments, and URD in each lane. **C** Growth curves of the WT, Δ*zur* mutant and Zur overexpression strains under control and 5 μM MV (Methyl Viologen) stress conditions. The x-axis indicates time and y-axis indicates OD_730_. **D** The levels of chlorophyll a in the WT, Δ*zur* mutant and Zur overexpression strains under control and 5 μM MV stress conditions. **E** Cultures of the WT, Δ*zur* mutant and Zur overexpression strains under control and 5 μM MV stress conditions. Error bars represent ± SEM (*n* = 3). *P < 0.05; **P < 0.01; ns, not significant

Zur proteins have been reported to enhance bacterial resistance to oxidative stress by regulating relative genes (Marin et al. 1998; Sein-Echaluce et al. 2015). The *sll0223* gene, which encodes NAD(P)H dehydrogenases NdhB, plays an important role in respiration, photosynthesis, and stress resistance in *Synechocystis* sp. PCC 6803 (Ogawa 1991; Mi et al. 2000; Hualing 2022; Thomas et al. 2001). Transcriptome analysis revealed that Zur upregulates the expression of the *sll0223* gene with a log_2_(Δ*zur*/WT) foldchange of -1.05. We validated the upregulation of the *sll0223* gene by Zur using qRT-PCR (Fig. 5A). Through EMSA, Zur was demonstrated to regulate *sll0223* by directly binding to its promoter region (Fig. 5B and Fig. S2G). To investigate the role of Zur in oxidative stress resistance, we evaluated the growth and photosynthetic pigment contents of the WT, Δ*zur* mutant, and Zur overexpression strains in liquid BG11 medium supplemented with 5 μM Methyl Viologen (MV). Under oxidative stress, both the WT and the overexpression strains showed notable growth advantage compared to the Δ*zur* mutant strain (Fig. 5C, E and Fig. S3B). Post-stress induction, the Δ*zur* mutant strain showed a significant reduction in chlorophyll a content compared to WT and overexpression strains (Fig. 5D) and the ROS levels in the mutant strains are significantly lower than those in the overexpressing strains (Fig. S6). These results suggest that Zur facilitates oxidative stress resistance by upregulating the expression of the *sll0223* gene in *Synechocystis* sp. PCC 6803.

### The expression of Zur influences biofilm formation and antibiotic resistance

**Fig. 6:**
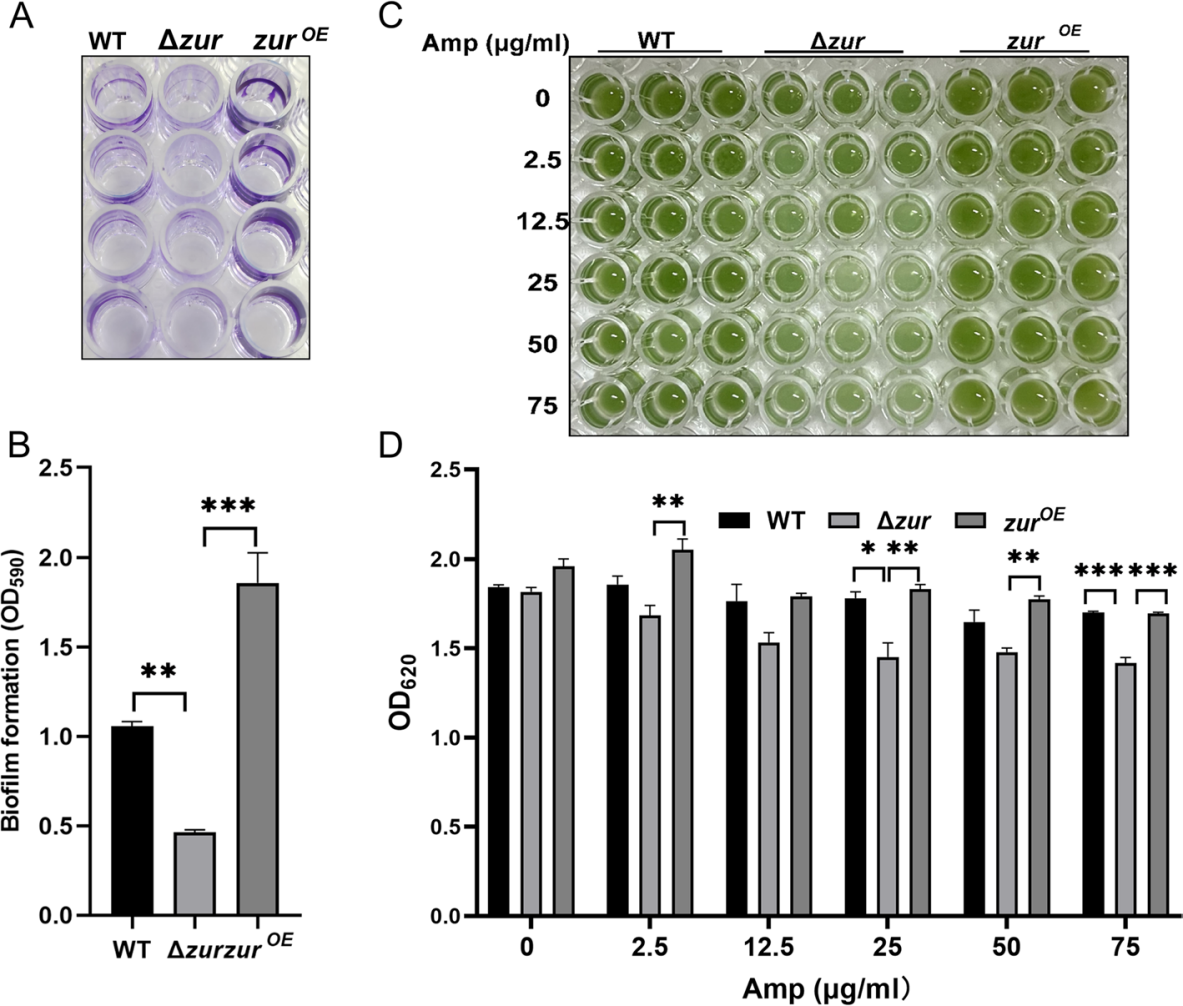
Zur influences the biofilm formation and antibiotics resistance. **A** and **B** Biofilm assays of the WT, Δ*zur* mutant, and Zur overexpression strains cultured in BG11 medium within a 96-well plate. **C** Photograph of the microtiter plate containing the WT, Δ*zur* mutant, and Zur overexpression strains with increasing concentrations of ampicillin. **D** Estimation of chlorosis by reading an OD_620_ of the microtiter plate. Error bars represent ± SEM (*n* = 3). *P < 0.05; **P < 0.01; ***P < 0.001

Previous studies have indicated that Zur not only plays a pivotal role in metal ion metabolism and resistance to oxygen and salt stress, but also impact biofilm formation and antibiotic resistance (Randazzo et al. 2020; Olivan-Muro et al. 2023). To investigate the effect of Zur on biofilm formation, we cultured the WT, Δ*zur* mutant and Zur overexpression strains cultured in BG11 medium for 7 days. The resulting biofilms were stained with 0.1% crystal violet and measured using a microplate spectrophotometer (Fig. 6A and B). The Δ*zur* mutant strain exhibited the least biofilm formation, markedly lower than both the WT and overexpression strains. Conversely, the overexpression strain showed the highest biofilm content, approximately four times that of the mutant strain and twice that of the WT strain. To evaluate the role of Zur in antibiotic resistance, we subjected the WT, Δ*zur* mutant, and Zur overexpression strains to varying concentrations of ampicillin in 96-well plates and measured the results using a spectrophotometer. As shown in Fig. 6C and D, the *Synechocystis* sp. PCC 6803 strain lacking Zur was more susceptible to antibiotic stress compared to the WT and overexpression strains. We also assessed the effect of other antibiotics, namely gentamicin (Gm), sulfamethoxazole (Smz), tetracycline (Tet), and chloramphenicol (Cm) in serial assays. However, there were no obvious differences in the growth patterns of the WT, mutant strains, and overexpression strains when exposed to these antibiotics (Fig. S5). In summary, Zur can enhance both biofilm formation and antibiotic stress resistance in *Synechocystis* sp. PCC 6803.

## Discussion

The function and structure of Zur have been studied in various bacterial species, including *Escherichia coli*, *Bacillus subtilis*, and *Yersinia pseudotuberculosis* etc., employing multiple techniques such as transcriptome sequencing, microarrays, ChIP-sequencing (ChIP-seq), proteome analysis, and EMSA (Cai et al. 2021; Shin and Helmann 2016; Hou et al. 2023). Multiple functions of Zur, including maintainance of metal ion homeostasis, motility and stresses resistance, have been identified. These roles exhibit similarity across species but also display diversity. For instance, in addition to its principal role in zinc uptake regulation, Zur can also modulate the expression of the type VI secretion system in *Y. pseudotuberculosis* and the cell wall development in *Bacillus subtilis* (Randazzo et al. 2020; Cai et al. 2021). However, there has been limited research on Zur in cyanobacteria. In *Anabaena* sp. PCC 7120, Zur was found to upregulate 262 genes and downregulate 143 genes through transcriptome analysis, thereby affecting various biological processes such as desiccation tolerance, antioxidant response, trehalose synthesis, and saccharide transfer (Olivan-Muro et al. 2023). In this study, we utilized RNA-seq analysis to identify 141 DEGs regulated by Zur in *Synechocystis* sp. PCC 6803 which are involved in multiple pathways. The accuracy of the transcriptomic data was subsequently validated through qRT-PCR, EMSA, and physiological functional assays, as previously reported (Xu et al. 2022; Wang et al. 2020). The expression levels of the DEGs from the transcriptomic data were determined via qRT-PCR to validated the reliability of the RNA-seq data. The Zur’s binding activities on the promoter sequences of various DEGs were assessed by EMSA, further reinforcing the conclusions drawn about Zur regulation from the RNA-seq data. Subsequently, a series of physiological functional tests, including metal ion, motility, stress tolerance, biofilm formation, and antibiotic resistance assays, were conducted to examine the regulatory functions of Zur in vivo based on the relative functional DEGs. Notably, the observed physiological functions were consistent with those of the genes regulated by Zur in the RNA-seq data, further bolstering the reliability of the transcriptomic analysis. This study elucidates the functions of Zur in metal ion homeostasis, motility, biofilm formation, salt stress, oxidative stress, and antibiotic stress resistance in *Synechocystis* sp. PCC 6803.

Zur and Fur can modulate motility by regulating the expression of flagellar genes in various bacteria including *E. coli*, *Pectobacterium odoriferum* and *Y. pseudotuberculosis* (Hou et al. 2023; Chen et al. 2023a; Gu et al. 2024a). However, cyanobacteria do not possess flagella, and predominantly exhibit gliding and twitching motility through pilus (Nakane 2023). The twitching motility in cyanobacteria relies on the rapid extension and retraction of Type IV pilus on the cell surface to achieve discontinuous, short-distance movement. This enables these bacteria to optimize light acquisition, nutrient uptake, and adaptation to environmental changes. This form of motility has been documented in *Synechocystis* sp. PCC 6803 and *Nostoc punctiforme* (Wilde and Mullineaux 2015). In *Myxococcus xanthus,* the *pilH* gene can influence type IV pilus biogenesis and social gliding motility (McBride 2001). The expression of the *pilG*, *pilH*, *pilI*, and *pilJ* genes was found to be more or less essential for pilus assembly, motility and the capacity for natural transformation with exogenous DNA by gene knockout analysis in *Synechocystis* sp. PCC 6803 (Yoshihara et al. 2002). We found that Zur positively modulates the expression of the *sll1291*-*sll1296* gene cluster corresponding to *pilG*, *pilH*, *pil, pilJ and cheA* homologous (Fig. 3). As previously reported, the disruption of any genes in *sll1291*-*96* cluster did not affect the phototactic motility (Bhaya et al. 2001), suggesting that the regulation of this gene cluster is likely not the primary factor by which Zur modulates the motility of *Synechocystis* sp. PCC 6803. Bacterial motility is not solely linked to flagella and pili but also regulated by second messengers such as c-di-GMP, cAMP, cGMP, and Ca^2^⁺(Opoku-Temeng and Sintim 2017; Varnum and Soll 1984; Liu et al. 2024; Kikuyama 2001). Prior research has indicated that Sycrp1, acting as a cAMP receptor protein (CRP), binds to cAMP and directly regulates the expression of genes involved in motility, as determined by DNA microarray analysis in *Synechocystis* sp. PCC 6803 (Yoshimura et al. 2002). In *P. aeruginosa*, the *pilJ* mutant strain exhibits a significant decrease in intracellular cAMP levels to 20% of the WT, and simultaneously loses its ability to perform twitching motility. Nevertheless, as long as adequate cAMP levels are maintained, the capacity for twitching motility in *P. aeruginos*a remains intact (Jansari et al. 2016). The DUF4114 domain in PilY1.1 potentially encompasses a conserved Ca^2+^-binding site. Mutations at this site lead to a reduction in motility, suggesting that the DUF4114 domain facilitates the binding of Type IV pili (T4P) to exopolysaccharides (EPS) through Ca^2^⁺binding, thereby enhancing motility in *M. xanthus* (Xue et al. 2022). We found that the CRP encoded by *sll1371* and the DUF4114 domain-containing protein that binds Ca^2+^, encoded by *sll0723*, are also positively regulated by Zur in this study. This may be a concomitant mechanism of Zur regulation of the motility of this cyanobacterium. Taken together, Zur can regulate pili-related genes as well as the expression of CRP and Ca^2+^-related genes to modulate cell motility in *Synechocystis* sp. PCC 6803.

Zur plays a pivotal role in maintaining the homeostasis of zinc ion and other metal ions in bacteria. The metabolism of zinc ions is intricate and multifaceted, involving numerous pathways. These include the zinc uptake systems ZnuABC, ZinT, AdcABC, and the zinc ion export systems such as *Z*itB and Zrf, along with zinc-binding outer membrane proteins TonB-dependent receptors in bacteria (Mikhaylina et al. 2018). The primary function of Zur is to regulate the expression of the ubiquitously distributed ZnuABC systems to maintain intracellular zinc ion homeostasis across various bacterial species including *E. coli*, *B. subtilis*, *Listeria monocytogenes*, *Yersinia pestis*, *Y. pseudotuberculosis*, and *Salmonella enterica* (Kandari et al. 2021; Petrarca et al. 2010; Sabri et al. 2009). Previous reports have indicated that Zur binds to the promoter region of *znuABC* in *Synechocystis* sp. PCC 6803 (Tottey et al. 2012)*.* In the present study, we demonstrated that Zur downregulates the expression of *znuABC* by directly binding to its promoter, thereby modulating the zinc ion concentration in *Synechocystis* sp. PCC 6803, which was consistent with previous reports in other species (Fig. 2). Furthermore, Zur also has the potential to influence iron absorption by regulating the corresponding genes. A putative cross-talk between the Zur and Fur regulatory network has been reported in *C. crescentus* (Mazzon et al. 2014). Our findings revealed that Zur could regulate the ferric uptake regulator protein Fur (Sll0567) (Fig. 2E and F), as well as ferric binding proteins such as Fe-S protein (Sll1348) and ferredoxin (Sll1584) (Table S2). This regulation subsequently modulates the iron concentration and even orchestrates the balance between zinc and ferric (Fig. 2D and G). These results not only confirmed the cross-talk between Zur and Fur regulatory network in *Synechocystis* sp. PCC 6803, but also underscored the multifunctionality of Zur and the complexity of metal ion regulatory network in bacteria.

Zur plays a multifaceted role in biological processes beyond maintaining metal ion homeostasis and motility. It also exhibits antioxidant, anti-salt, and antibiotic resistance properties, as well as influencing biofilm formation (Randazzo et al. 2020; Mikhaylina et al. 2018; Kim et al. 2021). The Zur mutant strain of *Acinetobacter baumannii* exhibited significantly reduced activities of superoxide dismutase (SOD) and catalase (CAT), leading to elevated levels of superoxide anion radicals (·O_2_^−^) and hydrogen peroxide (H_2_O_2_). This weakens the antioxidant capacity of the bacterium and heightens its susceptibility to multiple antibiotics, including colistin, gentamicin, rifampicin and tigecycline (Ajiboye et al. 2019). In *Anabaena* (*Nostoc*) sp. PCC 7120, Zur protects DNA and enhances cell survival under oxidative stress conditions (López-Gomollón et al. 2009). Moreover, in *B. subtilis* and *Paenibacillus polymyxa*, the expression of NADH dehydrogenase can regulate the intracellular NAD + /NADH ratio to resist extrinsic oxidative stress (Martín et al. 1996; Yu et al. 2019). In our RNA-seq results, Zur positively regulates the expression of NADH dehydrogenase subunit 2 NdhB (Sll0223) in *Synechocystis* sp. PCC 6803. Accordingly, the expression of the *sll0223* gene was significantly decreased in the Δ*zur* mutant strain, which was consistent with the decrease in antioxidant capacity and antibiotic resistance. Although Zur has been studied extensively for its function in oxidative stress, the role of Zur in osmotic stress resistance remains relatively understudied. In *Burkholderia insecticola* and *Bradyrhizobium diazoefficiens*, the deletion of the *otsA* gene results in a decrease in trehalose synthesis and increase susceptibility to osmotic stress induced by high salt or high sucrose levels (Lee et al. 2023; Ledermann et al. 2021). Transcriptomic analysis has also revealed that Zur positively regulates the expression of the *ostA* (*sll1566*) gene in *Synechocystis* sp. PCC 6803 by directly binding to its promoter region, thereby conferring tolerance to external high-salt stress. In addition to regulating the expression of related genes, bacteria can also counteract external pressures such as osmotic stress and antibiotics by modulating biofilm formation (Flemming et al. 2007). The increased susceptibility of the Zur mutant strain to oxygen, salinity, and antibiotics is directly linked to its compromised capacity to form biofilms. Conversely, the strain overexpressing Zur demonstrates the highest biofilm content and the greatest adaptability to external environmental conditions in *Synechocystis* sp. PCC 6803. In summary, Zur enhances both biofilm formation and the ability of *Synechocystis* sp. PCC 6803 to cope with various stresses, thereby improving its environmental adaptability.

In industrial production, bacteria encounter various stresses, including oxidative stress, salt stress, and antibiotics (Xu et al. 2023; Yang et al. 2021; Bucka-Kolendo and Sokołowska 2017). The zinc uptake regulator, Zur, can modulate the balance of zinc and iron ions, which serve as vital cofactors for superoxide dismutase, transcription factors, and other enzymes, playing key roles in cellular respiration and metabolism (Chen et al. 2023b; Bashir et al. 2016). By orchestrating the expression of Zur, the concentrations of metal ions and specific functional proteins in bacterial cells can be optimized, thereby enhancing the growth rate, metabolic efficiency, and even chemical production. Cellular motility also plays a pivotal role in industrial bioproduction, especially in the processes of biofilm formation and cell dispersion. Enhanced motility can aid cells in more efficient dispersion and nutrient searching, thereby improving production efficiency (Aroney et al. 2021; Ye et al. 2022). This study demonstrates that Zur significantly boosts cellular motility, indicating that the cyanobacteria with improved motility can be engineered by modulating Zur’s expression, thereby improving their dispersion ability and production efficiency in industrial fermentation. Biofilms hold substantial application value in bioremediation, wastewater treatment, and biocatalysis (Cheng et al. 2023; Wang et al. 2024; Maurya et al. 2023). The marked improvement in biofilm formation and stress tolerance facilitated by Zur indicates a promising application in chassis cells improvement for environmental remediation. Given this, this study has identified a potential functional element, Zur, that could be instrumental in enhancing the application of cyanobacteria.

## Conclusions

In conclusion, our findings elucidate the functional roles and underlying mechanisms of the transcriptional regulator Zur in *Synechocystis* sp. PCC 6803. Through transcriptomic analysis, we identified the global regulons of Zur. Zur not only collaborates with Fur to modulates metal ions homeostasis but also plays pivotal roles in cell motility, biofilm formation, and stress resistance. In particular, Zur enhances the strain’s resistance to oxygen stress, osmotic stress and antibiotic stress. This study highlights the comprehensive regulatory capacity of Zur and its significance for *Synechocystis* sp. PCC 6803 in environmental adaptation during growth.

## Materials and methods

### Culture conditions

The *Synechocystis* sp. PCC 6803 strain was sourced from the Freshwater Algae Culture Collection of the Institute of Hydrobiology, Chinese Academy of Sciences. The strains were cultured in liquid BG11 medium containing kanamycin and incubated at 30 °C under a light intensity of 60 µmol photons m^−2^ s^−1^, provided by fluorescent lamps, with shaking at 200 rpm (Cho et al. 2021). Solid BG11 medium was supplemented with 1.5% agar. For growth assays, cells were collected at the stationary phase and resuspended in fresh liquid BG11 medium containing kanamycin antibiotic to an OD_730_ of 0.08. Liquid BG11 medium was supplemented with 5 μM MV, 4% NaCl (Mass/Volum), and 8 μM ZnCl_2_, and the OD_730_ was measured every 24 h using a microplate spectrophotometer (Biotek Instruments, USA) during the stress experiments.

### Construction of mutants

Using the genome of *Synechocystis* sp. PCC 6803 as a template, the *zur* gene, along with its 500 bp upstream and downstream regions, was amplified by PCR using the primer pairs *sll1937*F/*sll1937*R, *sll1937*UF/*sll193*7UR, and *sll1937*DF/*sll1937*DR. Additionally, the kanamycin resistance gene (*km*^*r*^) and *biPpsbA*_2_ gene were amplified using the primer pairs *Kana*F/*Kana*R and *ppsb*F/*ppsb*R (Table S1). Subsequently, the *zur* upstream, *biPpsbA*_2_, *zur*, *km*^*r*^, and *zur* downstream gene fragments were overlapped to form contiguous sequences by PCR, and then ligated into the pMD19T vector to construct pMD19T-*zur*-overexpression plasmid. Similarly, the *zur* upstream, *biPpsbA*_2_, *km*^*r*^, and *zur* downstream gene fragments were overlapped and ligated into pMD19T to generate pMD19T-*zur*-delete plasmid (Vachiranuvathin et al. 2022). Both plasmids were transformed into *Synechocystis* sp. PCC 6803 strains to produce Zur mutant and overexpression strains. The wild-type (WT) strain of *Synechocystis sp.* PCC 6803 was also modified to confer kanamycin resistance using a similar approach ( Fig. S1).

### Purification of Zur protein

The pET28a-*zur* plasmid was constructed by digesting the pET28a vector and the *zur* gene amplified with primer pairs s*ll1937-NdeI*F*/sll1937*-*XhoIR* with *NdeI* and *XhoI* enzymes*,* and then ligating them with T4 ligase (Accurate Biotech, Hunan, China) (Table S1). This plasmid was then transformed into the *E. coli* BL21(DE3) strain. When this bacterium was grown to logarithmic phase at 37 °C, Zur protein expression was induced by adding 0.25 mM IPTG to the culture overnight. After harvesting the bacteria, cells were lysed and purified with His-Bind Ni–NTA resin (Novagen, Madison, USA) (Gu et al. 2024b). The purity and concentration of proteins were measured by 12% SDS-PAGE and NanoDrop ND-1000 spectrophotometer (ThermoFisher Scientific, USA), respectively.

### RNA-seq experiment

When the WT and Δ*zur* strains that were grown in BG11 culture reached log phase, cells were collected by centrifugation at 4500 rpm, with three replicates for each group. RNA isolation, cDNA library construction and sequencing, RNA-seq analysis were commissioned by Sangon with the Illumina HiSeq Xten platform (Shanghai, China). Then, DESeq2 was used to identify diferentially expressed genes (DEGs) with |log_2_ (fold change) |> 1 and Q-value < 0.05 as screening standards. Cluster of Orthologous Groups of proteins (COG) enrichment was conducted using cluster Profler Package in R (Hu et al. 2021).

### Detection of ROS

Cells were grown in BG11 medium, 2 ml of cell culture was collected into a centrifuge tube, centrifuged, washed twice with fresh BG11, and finally fixed in 1 ml. The medium was supplemented with 10 μM DCFH-DA, incubated in the dark for 30 min at 37 °C (Hu et al. 2022). Then the medium was transferred to opaque black 96-well plates (Costar, USA) to determine the fluorescence at 488 nm excitation and 525 nm emission by Spectra Max M2 microplate reader (Molecular Devices, USA) (Hu et al. 2021).

### Measurement of photosynthetic pigment content

2 ml cultures of *Synechocystis* sp. PCC 6803 were collected and centrifuged at 13,000 rpm for 10 min. The precipitate was resuspended with 1 ml of N,N-dimethylformamide solution and centrifuged at 13,000 rpm for 10 min. The supernatant was taken to determine OD_461_, OD_625_, and OD_664_ by spectrophotometry, respectively, and calculated the total carotenoid and chlorophyll a contents according to the formulas (Fang et al. 2017; Hu et al. 2022).

$$Carotenoids (\mu g/ml) = (OD461 - 0.046 \times OD664) \times 4$$

$$Chlorophyll a (\mu g/ml) = 12.1 \times OD664 - 0.17 \times OD625$$

### Quantitative real-time PCR (qRT-PCR) analysis

The *Synechocystis* sp. PCC 6803 strains were cultured in BG11 medium with 200 rpm until OD_730_ reached 1.0, and cells were collected by centrifugation. Total RNA was extracted with the MolPure® Bacteria RNA Kit (Yeasen, Shanghai, China) and cDNA was obtained by EasyScript One-step gDNA Removal and cDNA Synthesis SuperMix (TransGen Biotech, Beijing, China) following the manufacturers’ instructions. The qRT-PCR was conducted with the SYBR Green *Pro Taq* HS Premix (Accurate Biotech, Hunan, China) with at least three replicates per group. The *rnpb* was used as an internal reference gene for the qPCR analysis (Ikeuchi and Tabata 2001).

### Electrophoretic mobility shift assay (EMSA)

The 450 bp DNA probes and 200 bp URD were amplified by PCR and purified using a 1% agarose gel. Binding assays were conducted as previously described. Briefly, reactions were performed with a mixture composed of the 1 × Binding Buffer (10 mM Tris, 50 mM KCl, 1 mM DTT, and 5% Glycerol), Zur protein, 30 ng of DNA, BSA in the absence Zur protein and incubated at room temperature for 20 min. Samples were then separated on a 6% polyacrylamide native gel at 4℃ in Tris–borate-EDTA buffer. The gel was stained with SYBR Safe DNA gel stain and imaged using a fluorescence imaging system (Tanon 5200Multi, Tanon, China) (Gu et al. 2024a).

### Determination of intracellular ion content

Methods for determination of cellular iron content were described in previous studies (Si et al. 2017). Collect the cells cultured for 10 days in 30 ml of BG11 liquid medium supplemented with 8 µM zinc ions. Wash the cells twice with 10 mM EDTA-Na_2_ and twice with ddH₂O to remove extracellular metal ions. Then the bacterial precipitate was lysed with Bugbuster (Novagen, USA) according to the manufacturer’s instructions, and then centrifuged and the supernatant was taken and added to 2% HNO_3_ for overnight digestion. Metal contents were determined using ICP-MS (ThermoFisher Scientific, USA), and the results were normalized using protein content.

### Biofilm formation assay

When the strains grew to logarithmic phase, the culture was adjusted to OD_730_ = 0.3 with BG11 medium, and 200 μL of culture was taken into 96-well plates and incubated at 30℃ for 7 days. Then the culture was discarded and washed twice with ddH_2_O to remove impurities. Biofilms were stained with 0.1% crystal violet for 30 min, then dissolved in 70% ethanol, and finally OD_590_ was measured using a microplate photometer (Olivan-Muro et al. 2023; Kera et al. 2018).

### Motility assays

The cyanobacterial cells were collected in the late logarithmic phase and spotted into solid BG11 plate containing 0.8% (wt/vol) agar, 10 mM HEPES (PH 7.5), 5 mM glucose, and 0.3% (wt/vol) sodium thiosulfate. The plates were then placed in an opaque box with a 3 cm wide slit and incubated for 10 days at 28 °C, with a light intensity of 60 μmol photons m^−2^ s^−1^ on one side of the slit (Song et al. 2018; Savakis et al. 2012). The distance from the inoculation point to the colony edges nearest (D1) and furthest (D2) from the light source was measured, and the response index (RI) was calculated using the formula: RI = D1 / (D1 + D2) (Zhang et al. 2020).

### Antibiotic tolerance assay

Cyanobacterial cells with OD_730_ = 0.9 were collected, washed twice with fresh BG11 and resuspended. Ampicillin antibiotic was added to make the final concentration of 0, 2.5, 12.5, 25, 50, 75 μg/ml. Then, 200 μL of culture was taken into 96-well plate and incubated under light for 7 days. Chlorosis was estimated by reading the absorbance at 620 nm using microplate photometer (Sein-Echaluce et al. 2015).

### Statistical analysis

Statistical significance was performed using an One-way ANOVA with GraphPad Prism Software (GraphPad Software, San Diego, California, USA). Error bars represent ± SEM. *P < 0.05; **P < 0.01; ***P < 0.001; ****P < 0.0001; n.s., not significant.

## Supplementary Information

Supplementary Material 1.

## Data Availability

The RNA-seq data are available in the NCBI Sequence Read Archive database (SRA accession: PRJNA1174049). Other datasets generated for this study are included in the article/Supplementary Information.

## Abbreviations

RNA: Ribonucleic acid
DNA: Deoxyribonucleic acid
WT: Wide Type
Zur: Zinc uptake regulator
Fur: Ferric uptake regulator
KEGG: Kyoto Encyclopedia of Genes and Genomes
COG: Clusters of Orthologous Groups of proteins
T4P: Type IV pili
GGPS: Glucosylglycerol-phosphate synthase
MV: Methyl Viologen
ChIP-seq: ChIP-sequencing
CRP: CAMP receptor protein
CAT: Catalase
H_2_O_2_: Hydrogen peroxide
RI: The response index
IPTG: Isopropyl β-D-Thiogalactopyranoside
PCR: Polymerase Chain Reaction
qRT-PCR: Quantitative Real-time PCR
EMSA: Electrophoretic Mobility Shift Assay
ROS: Reactive Oxygen Species
EPS: Exopolysaccharides
SOD: Superoxide dismutase
·O_2_^−^: Superoxide anion radicals
BG11: BG11 Medium for Blue Green Alga
URD: Unrelated-DNA fragment
BSA: Bovine Serum Albumin
ICP-MS: Inductively coupled plasma-Mass Spectrometry
SDS-PAGE: Sodium dodecyl sulfate polyacrylamide gel electrophoresis
