# Supplementary material for: Functional investigation of Zur in metal ion homeostasis, motility and multiple stresses resistance in cyanobacteria Synechocystis sp. PCC 6803
Source: Stress Biol. 2025 May 7;5(1):32. doi: 10.1007/s44154-025-00224-x (PMC12058595; doi:10.1007/s44154-025-00224-x)
Supplement: Supplementary file 1 — Supplementary Material 1. [file 44154_2025_224_MOESM1_ESM.docx]

**Supplementary material**

**Functional investigation of Zur in metal ion homeostasis, motility and multiple stresses resistance in cyanobacteria**

***Synechocystis* sp. PCC 6803**

**This file includes:**

Supplementary Figure 1

Supplementary Figure 2

Supplementary Figure 3

Supplementary Figure 4

Supplementary Figure 5

Supplementary Figure 6

Supplementary Table 1

Supplementary Table 2

**
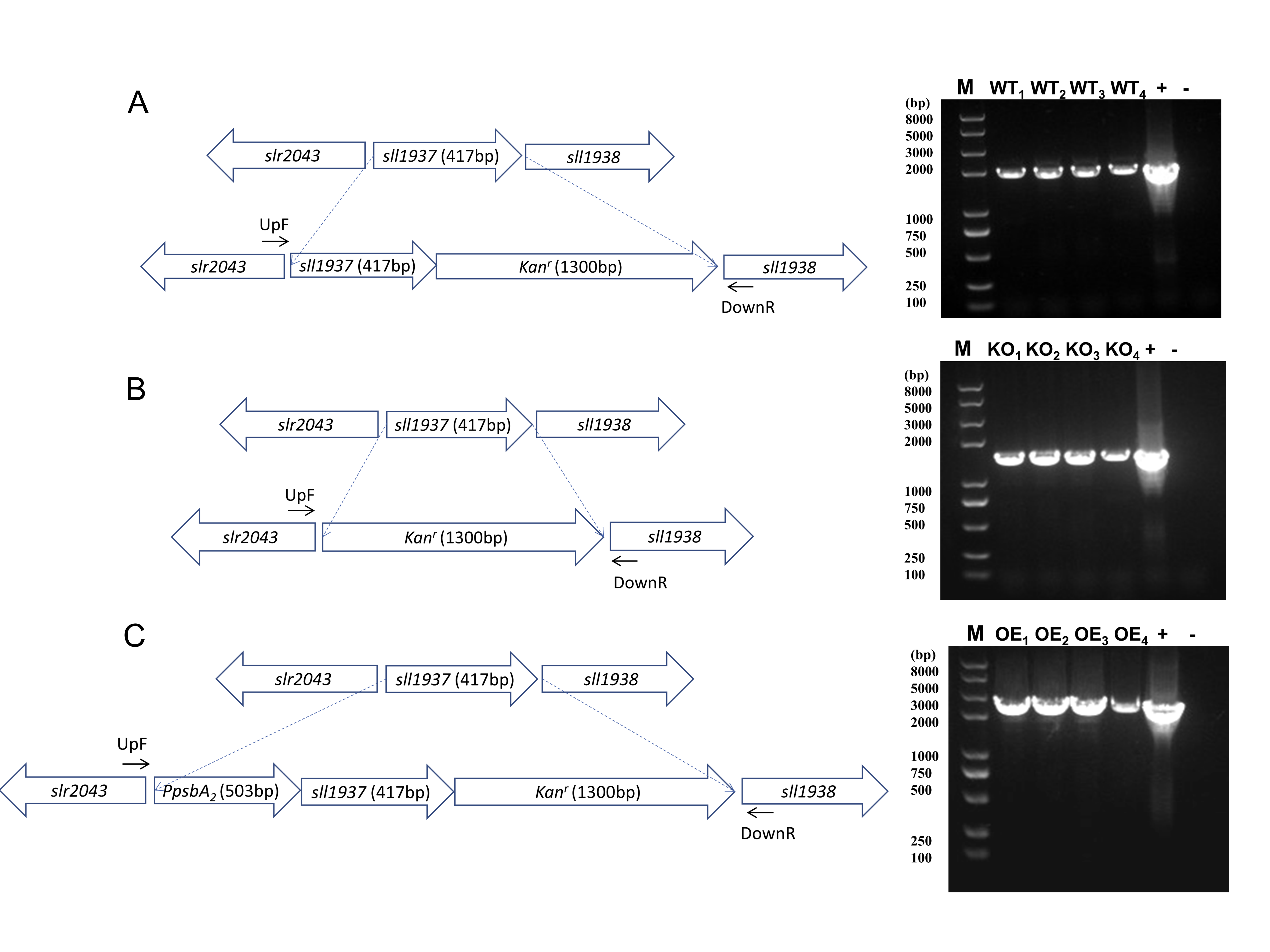
Fig. S1. Varification of the transgenic strains by** **colony PCR.** The schematics of manipulated gens in the WT (A), *zur* knock-out (B) and overexpression (C) strain, and the results of PCR using respective primers.

**
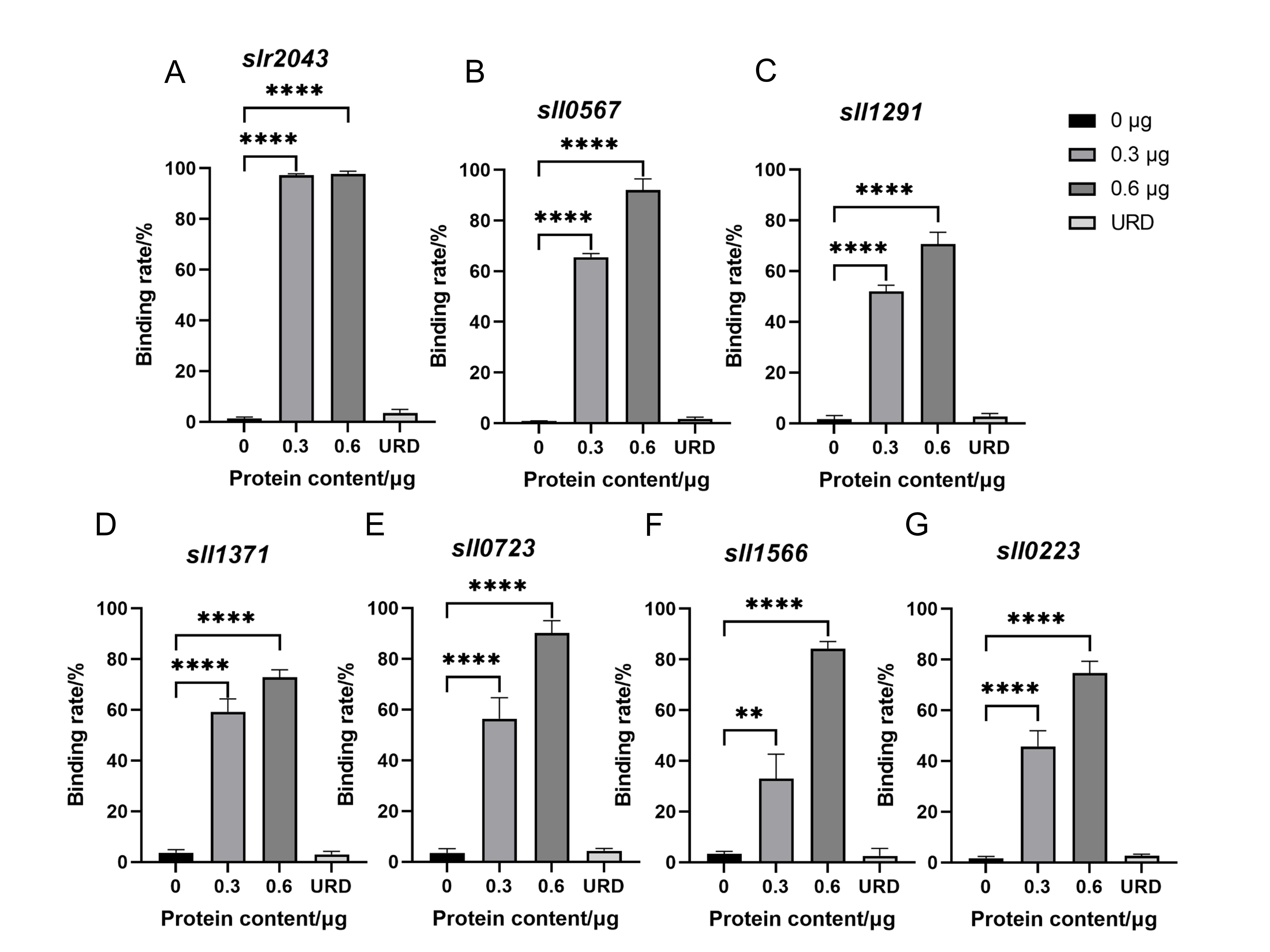
**

**Fig. S2. The quantification of the EMSA images.** Binding rate levels of *slr2043*, *sll0567*, *sll1291*, *sll1371*, *sll0723*, *sll1566* and *sll0223* promoters by Zur. Mean values with standard deviations (error bars) from at least three repeats are shown. **P < 0.01; ****, P<0.0001.


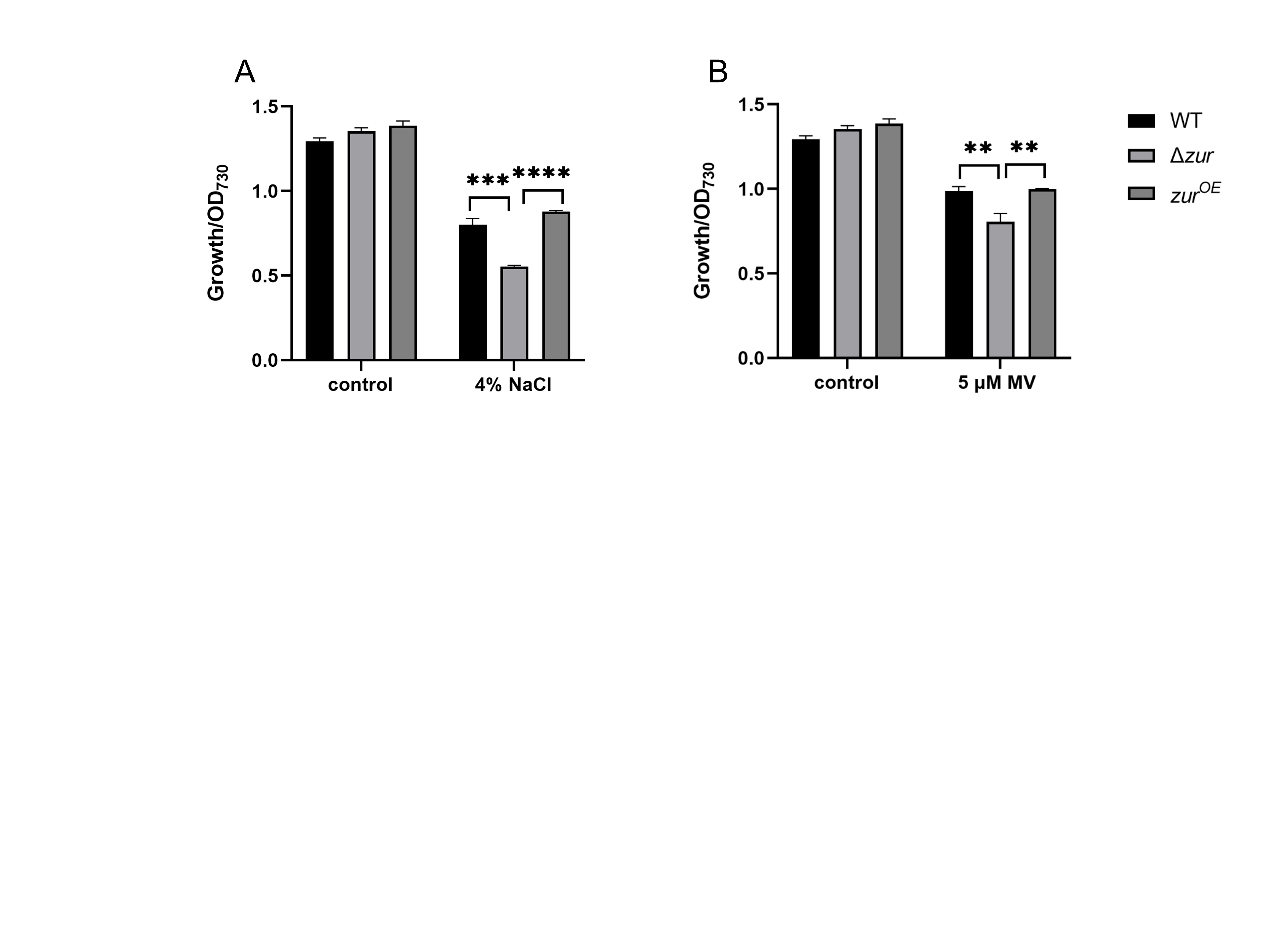


**Fig. S3. The quantification of Fig. 4G and 5E.** (A) The quantification of cultures of the WT, Δ*zur* mutant and Zur overexpression strains under control and 4% NaCl stress conditions.（B）The quantification of cultures of the WT, Δ*zur* mutant and Zur overexpression strains under control and 5 μM MV stress conditions. Error bars represent ± SEM (n=3). **P < 0.01; ***P < 0.001; ****P < 0.0001; ns, not significant.


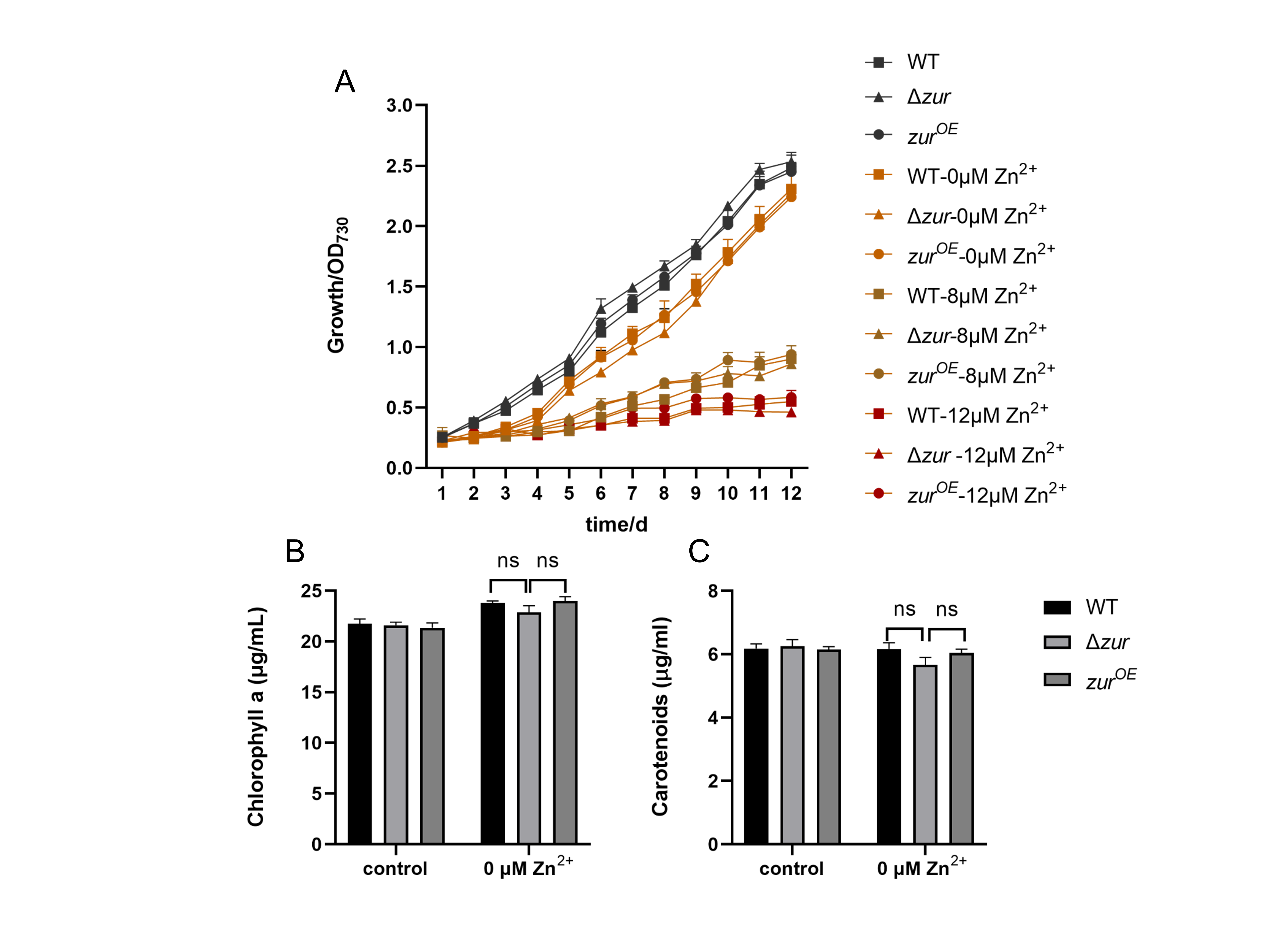


**Fig. S4. Growth under different zinc ion conditions.** (A) Growth curve of the WT, Δ*zur* mutant and Zur overexpression strains under control and 0, 8, 12 μM Zn^2+^. (B) chlorophyll a levels of WT, Δ*zur* mutant and Zur overexpression strains under control and 0 μM Zn^2+^. (C) Carotenoids of WT, Δ*zur* mutant and Zur overexpression strains under control and 0 μM Zn^2+^. Error bars represent ± SEM (n=3). ns, not significant.


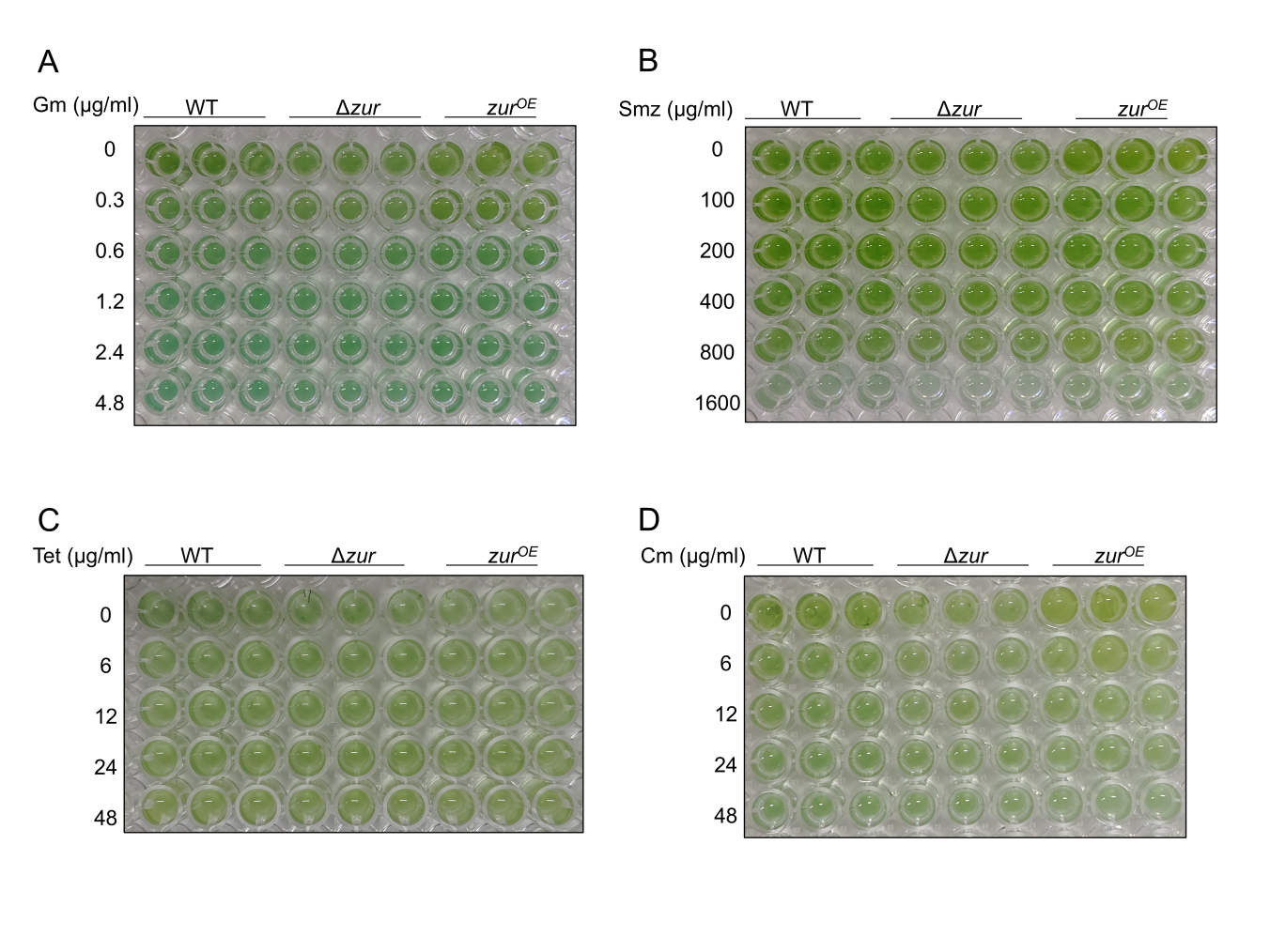


**Fig. S5. Growth under different antibiotic stress conditions.**  Photograph of the microtiter plate containing the WT, Δ*zur* mutant, and Zur overexpression strains with gentamicin (Gm) (A), sulfamethoxazole (Smz) (B), tetracycline (Tet) (C), and chloramphenicol (Cm) (D). The culture of strains was taken into 96-well plate and incubated for 7 days.


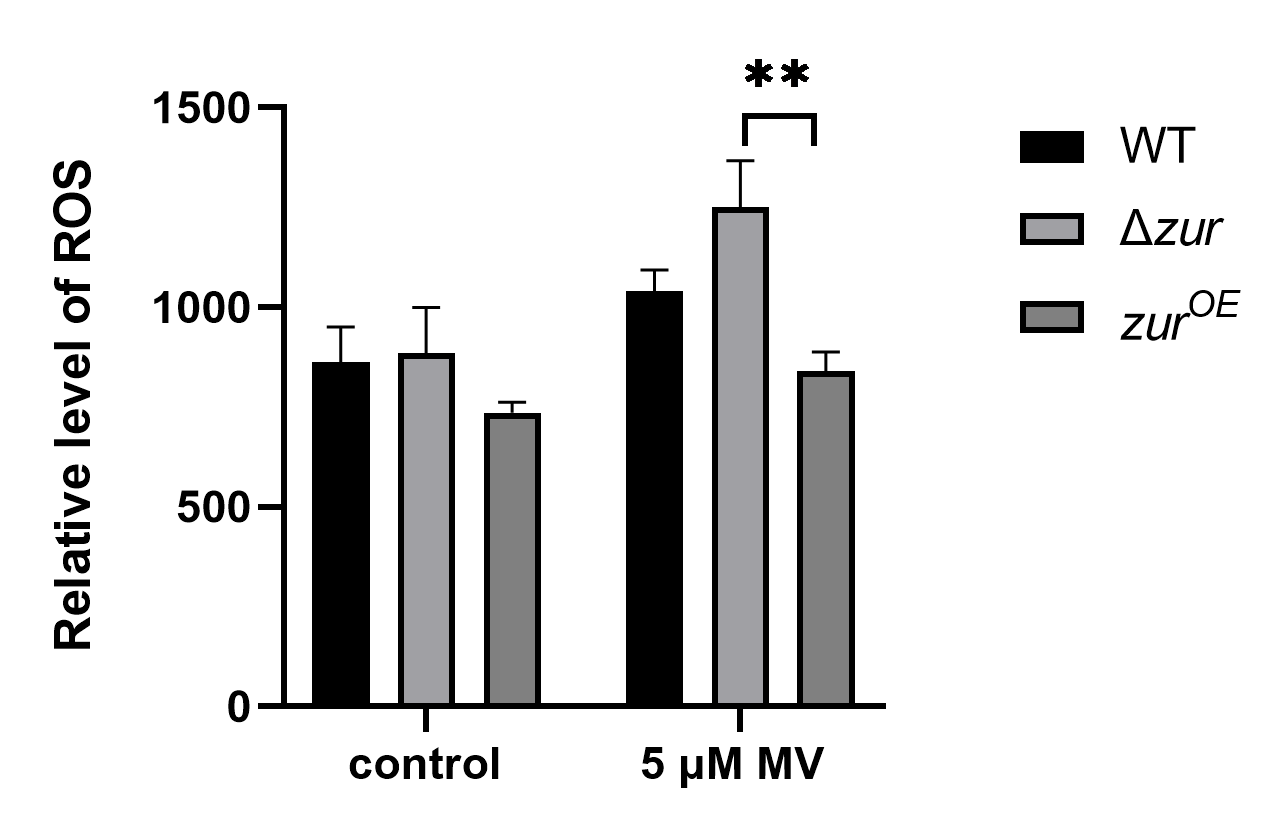


**Fig. S6. The relative levels of ROS in the WT, Δ*zur* mutant and Zur overexpression strains under control and 5 μM MV stress conditions**. Error bars represent ± SEM (n=3). **P < 0.01.

**Table S1.** Bacterial strains, plasmids and primers used in this study.

| **Strain, plasmid or primer** | **Relevant characteristics** | **Reference** |
| --- | --- | --- |
| ***E.coli*** |  |  |
| BL21(DE3) | Host for expression vector pET28a | Novagen |
| ***Synechocystis* sp. PCC 6803** |  |  |
| WT | Wild-type *Synechocystis* sp. PCC 6803, Km^r^ | Zheng et al.2023 |
| Δ*zur* | *zur* deleted in *Synechocystis* sp. PCC 6803, Km^r^ | This study |
| *Zur ^OE^* | Δ*zur* completed with *biPpsbA2-zur* in Δ*zur* in *Synechocystis* sp. PCC 6803, Km^r^ | This study |
| **Plasmids** |  |  |
| pMD19-T | Cloning vector, ColE1 replicon, Amp^r^ | Takara |
| pET28a | Expression vector with N-terminal hexahistidine affinity tag, Km^r^ | Novagen |
| pET28a*-zur* | *zur* in pET28a for Zur protein expression and purification, Km^r^ | This study |
| pMD19T-*zur*-overexpress | *zur* in pMD19T for construct Zur overexpression strain of *Synechocystis* sp. PCC 6803, Amp^r^ | This study |
| pMD19T-*zur*-delete | *zur* in pMD19T for construct Δ*zur* mutant strain of *Synechocystis* sp. PCC 6803, Amp^r^ | This study |
| **Primer** | **5’-3’ sequence** | **Function** |
| *sll1937*F | ACAAATACATAAGGAATTATAACCACGCGT  ATGAGTCTTCCCACTCCTTCCCTTG | For the construction of strains |
| *sll1937R* | GGAAAACCCTGGCGTTACCCAACTAGGCC  TCTAATCACTTCCTTTGGCACAAAGTTG |  |
| *sll1937*UF | CCTAATTCTTGATTTAACCGCTCT |  |
| *sll1937*UR | CTGGGAAAACCCTGGCGTTACCCAACTAG  GCCTGACAAGGTGGAGGAATTATGCAATG |  |
| *sll1937*DF | CATACGAGCCGGAAGCATAAAGTGAGATC  TAATTGAAGTTAACCAAAATAAGAATAG |  |
| *sll1937*DR | ACAATCCAAACAACAAGCCCGGCT |  |
| *Kana*F | AGGCCTAGTTGGGTAACGCCAG |  |
| *Kana*R | AGATCTCACTTTATGCTTCCGGC |  |
| *ppsb*F | ATTGCATAATTCCTCCACCTTGTCAGGCCT  TGCGGCTTTAGCGTTCCAGTGGATA |  |
| *ppsb*R | ACGCGTGGTTATAATTCCTTATGTA |  |
| *sll1937-NdeIF* | GGAATTCCATATGAGTCTTCCCACTCCTTCCCT |  |
| *sll1937*-*XhoI*R | CCGCTCGAGCTAATCACTTCCTTTGGCA |  |
| *slr2043* F | TTCCCGCAGTTTGGCGAAGAGGG | For EMSA |
| *slr2043* R | ACTCACCGTAATATCCATGGCATCTGC |  |
| *sll0567* F | CAAAACCCCCTCAGAAATGGCT |  |
| *sll0567* R | GTAACTCCTCGGCACTGAGATGTT |  |
| *sll1291* F | TTTGAACTCATTAAACGGAGCAAACAGTT |  |
| *sll1291* R | GTTGATCACTTTGCTATGGGAACAATGCA |  |
| *sll1371* F | GCCAACCAAGTTATTCTCCTCGAACA |  |
| *sll1371* R | ACTAATACTACTCAATTCTCGGTGGG |  |
| *sll0723* F | GATTTAGGTTCAGCAAAATGGCCATCTAC |  |
| *sll0723* R | AGTGATTGAGGTAAAATCCAAATTTTTGGA |  |
| *sll1566* F | GTAAATTTCCAGAGGATACCCCCAACTCCA |  |
| *sll1566* R | ATCAGCGGTCTCCAAAATCAAGAATCAAAC |  |
| *sll0223* F | GATAGGGAAAAGGGGCTGAGGCAATTT |  |
| *sll0223* R | CAATCAAATCAACAATTAGCACAAGGAGGAG |  |
| *slr2043*F | cacgatgaccatagccacga | For qRT-PCR |
| *slr204*R | caccaaggtaggggacaacc |  |
| *sll0567*F | tgtcagctaaccgtcaccac |  |
| *sll0567*R | ccaaggaaatactgcgggtg |  |
| *sll1294*F | agttggacagtgacggcatt |  |
| *sll1294*R | gttggggaatagcggggatt |  |
| *sll1296*F | tggatgaagtgttgccccaa |  |
| *sll1296*R | gtcaacaatgccgagcgatc |  |
| *sll1371*F | ggctgagggaagcagaaagt |  |
| *sll1371*R | tcaattctcggtggggcaaa |  |
| *sll0723*F | ggtggctatgatccagtgca |  |
| *sll0723*R | gccgctcgatatgaaacagc |  |
| *sll1566*F | tttttccaccacaccccctt |  |
| *sll1566*R | aaccacagagatcacaggcc |  |
| *sll0223*F | gcttttctttcggtgggctc |  |
| *sll0223*R | gcggtgaaaataacgtgcca |  |
| *rnpB*F | tttagaaaacagcaaccagt |  |
| *rnpB*R | ggcaggaaaaagaccaacct |  |

Km^r^ and Amp^r^ represent resistance to kanamycin and Ampicillin at 50μg ml^-1^.

**References：**

Zheng Y, Xue C, Chen H, Jia A, Zhao L, Zhang J, Zhang L, Wang Q*.* 2023*.* Reconstitution and expression of *mcy* gene cluster in the model cyanobacterium *Synechococcus* 7942 reveals a role of MC-LR in cell division*.* New Phytol 238:1101-1114.

**Table S2.** DEGs identified between the Δ*zur* mutant and the WT by RNA-seq in *Synechocystis* sp. PCC 6803.

| **Functional group** | **Locus tag** | **Gene**  **id** | **GeneName** | **GeneDescription** | **log2(Δ*zur*/WT)** | **Pvalue(WT-vs-Δ*zur*)** | **Qvalue(WT-vs-Δ*zur*)** |
| --- | --- | --- | --- | --- | --- | --- | --- |
|  | sll1937 | SGL_RS04615 | Zur | Fur family transcriptional regulator | -19.10661405 | 000000000000000243 | 0000000000000495 |
| Energy production and conversion | sll1085 | SGL_RS11080 | glpD | glycerol-3-phosphate dehydrogenase | -2.103829607 | 1.97E-15 | 3.21E-13 |
|  | sll1023 | SGL_RS03770 | sucC | succinate--CoA ligase subunit beta | 1.675449902 | 0.000255597 | 0.003328896 |
|  | sll1348 | SGL_RS06980 | queG | tRNA epoxyqueuosine(34) reductase QueG | 1.214394967 | 0.001594232 | 0.013805371 |
|  | sll1584 | SGL_RS05195 | SGL_RS05195 | ferredoxin | 1.081114197 | 0.005014513 | 0.032840578 |
|  | sll0223 | SGL_RS02610 | ndhB | NAD(P)H-quinone oxidoreductase subunit N | -1.046727767 | 4.63E-14 | 5.38E-12 |
| Amino acid transport and metabolism | sll2001 | SGL_RS09430 | lap | leucyl aminopeptidase | 1.069625253 | 0.003198844 | 0.023998704 |
|  | sll0573 | SGL_RS09430 | arcC | carbamate kinase | -1.23821966 | 0.001209497 | 0.011187846 |
| Cell motility | sll1296 | SGL_RS05075 | CheA | hybrid sensor histidine kinase/response regulator | -1.187525091 | 0.000000524 | 0.0000198 |
|  | sll0858 | SGL_RS08155 | SGL_RS08155 | Spy/CpxP family protein refolding chaperone | 2.615378647 | 1.44E-19 | 5.87E-17 |
|  | sll1294 | SGL_RS05080 | pilJ | HAMP domain-containing methyl-accepting chemotaxis protein | -1.178224299 | 8.51E-10 | 6.02E-08 |
|  | sll1695 | SGL_RS07940 | hofG | type II secretion system protein | 1.203142572 | 0.0000746 | 0.001239739 |
| Inorganic ion transport and metabolism | sll0858 | SGL_RS08155 | SGL_RS08155 | SulP family inorganic anion transporter | 2.615378647 | 1.44E-19 | 5.87E-17 |
|  | sll0834 | SGL_RS15100 | SGL_RS15100 | SulP family inorganic anion transporter | -1.312649393 | 8.33E-08 | 0.00000377 |
|  | sll0450 | SGL_RS17955 | norB | nitric-oxide reductase large subunit | -1.340668173 | 0.000377244 | 0.004389862 |
| Secondary metabolites biosynthesis/ transport and catabolism | ssl2084 | SGL_RS05670 | acpP | acyl carrier protein | 1.085109759 | 0.00000807 | 0.000208569 |
|  | sll0723 | SGL_RS17765 | SGL_RS17765 | DUF4114 domain-containing protein | -2.067127102 | 3.15E-13 | 3.42E-11 |
| Nucleotide transport and metabolism | sll1258 | SGL_RS10020 | dcd | dCTP deaminase | 1.475303011 | 0.002903769 | 0.022194065 |
| Lipid transport and metabolism | sll1510 | SGL_RS04070 | SGL_RS04070 | sterol desaturase family protein | 1.743591557 | 0.000316877 | 0.003908144 |
|  | ssl2084 | SGL_RS05670 | acpP | acyl carrier protein | 1.085109759 | 0.00000807 | 0.000208569 |
|  | sll1752 | SGL_RS04445 | SGL_RS04445 | 1-acyl-sn-glycerol-3-phosphate acyltransferase | 1.768441881 | 6.03E-13 | 5.77E-11 |
|  | sll1848 | SGL_RS12400 | SGL_RS12400 | lysophospholipid acyltransferase family protein | -1.153568306 | 0.000527853 | 0.005544159 |
| Carbohydrate transport and metabolism | sll1566 | SGL_RS11085 | ggpS | glucosylglycerol-phosphate synthase | -2.251230017 | 2.95E-18 | 9.61E-16 |
|  | sll1479 | SGL_RS17560 | pgl | 6-phosphogluconolactonase | -1.634707366 | 6.45E-08 | 0.00000318 |
|  | sll1538 | SGL_RS11500 | bgl | beta-glucosidase | -1.358159513 | 0.001076676 | 0.010315033 |
|  | sll1306 | SGL_RS07380 | SGL_RS07380 | polysaccharide deacetylase family protein | -1.106517976 | 0.002224712 | 0.01792986 |
|  | sll1374 | SGL_RS10585 | melB | MFS transporter | 1.369745185 | 4.2E-10 | 3.35E-08 |
| Cell cycle control cell division chromosome partitioning | sll0252 | SGL_RS08915 | SGL_RS08915 | SpoIID/LytB domain-containing protein | 1.311365176 | 0.002120171 | 0.017344918 |
|  | sll1681 | SGL_RS02975 | SGL_RS02975 | hypothetical protein | 1.48388829 | 0.001454521 | 0.012831689 |
| Translation, ribosomal structure and biogenesis | sll0320 | SGL_RS13240 | rnd | ribonuclease D | 1.167182422 | 0.0000151 | 0.000333213 |
|  | sll0495 | SGL_RS16775 | asnS | asparagine--tRNA ligase | 2.118755539 | 1.45E-17 | 3.94E-15 |
|  | sll1865 | SGL_RS12270 | prfB | peptide chain release factor 2 | 1.09977684 | 0.00191789 | 0.01601192 |
|  | ssl1784 | SGL_RS03445 | rpsO | 30S ribosomal protein S15 | 1.492891062 | 0.00490769 | 0.032478535 |
|  | sll1110 | SGL_RS18110 | prfA | peptide chain release factor 1 | 1.355143334 | 0.000059 | 0.001033021 |
|  | sll1244 | SGL_RS10150 | rplI | 50S ribosomal protein L9 | 1.122297142 | 0.000156636 | 0.002236871 |
|  | sll0996 | SGL_RS04845 | miaB | tRNA (N6-isopentenyl adenosine(37)-C2)-methylthiotransferase MiaB | 1.160137188 | 0.000690246 | 0.00702325 |
|  | sll1740 | SGL_RS06230 | rplS | 50S ribosomal protein L19 | 1.070106352 | 0.001525087 | 0.013277227 |
|  |  | SGL_RS19005 | rpsU | 30S ribosomal protein S21 | 1.384668763 | 0.001793928 | 0.015132199 |
| Signal transduction mechanisms | sll1296 | SGL_RS05075 | CheA | hybrid sensor histidine kinase/response regulator | -1.187525091 | 0.000000524 | 0.0000198 |
|  | sll0858 | SGL_RS08155 | SGL_RS08155 | Spy/CpxP family protein refolding chaperone | 2.615378647 | 1.44E-19 | 5.87E-17 |
|  | sll1294 | SGL_RS05080 | pilJ | HAMP domain-containing methyl-accepting chemotaxis protein | -1.178224299 | 8.51E-10 | 6.02E-08 |
|  | sll0790 | SGL_RS16105 | SGL_RS16105 | ATP-binding protein | 1.910717375 | 0.00000301 | 0.000086 |
|  | sll0789 | SGL_RS16110 | OmpR | response regulator transcription factor | 1.632644831 | 0.00000105 | 0.0000355 |
|  | sll1895 | SGL_RS10225 | SGL_RS10225 | EAL domain-containing protein | 1.349544928 | 0.005989562 | 0.038089871 |
|  | ssl0564 | SGL_RS12615 | SGL_RS12615 | helix-turn-helix transcriptional regulator | 1.003936844 | 0.003889093 | 0.027648225 |
| Coenzyme transport and metabolism | sll0099 | SGL_RS15745 | cbi | bifunctional cobalt-precorrin-7 (C(5))-methyltransferase/cobalt-precorrin-6B (C(15))-methyltransferase | 1.399090271 | 0.002142323 | 0.017438512 |
|  | sll1185 | SGL_RS03345 | hemF | oxygen-dependent coproporphyrinogen oxidase | 1.308266099 | 0.0000649 | 0.001111748 |
| Posttranslational modification/protein turnover/chaperones | sll1680 | SGL_RS02980 | msrB | peptide-methionine (R)-S-oxide reductase MsrB | 1.030750656 | 0.007377389 | 0.043515903 |
|  | sll0897 | SGL_RS16870 | dnaJ | molecular chaperone DnaJ | 1.064750161 | 0.006829957 | 0.041644833 |
|  | sll1898 | SGL_RS10200 | SGL_RS10200 | heme A synthase | -1.681087576 | 6.89E-16 | 1.25E-13 |
| Defense mechanisms | sll8049 | SGL_RS01125 | SGL_RS01125 | type I restriction endonuclease subunit R | -1.326077085 | 0.0000179 | 0.000374532 |
|  | sll1725 | SGL_RS06320 | ABC transporter | ABC transporter ATP-binding protein | 2.37204307 | 3.73E-13 | 3.8E-11 |
| Transcription | sll0856 | SGL_RS08165 | rpoE | sigma-70 family RNA polymerase sigma factor | 2.210760266 | 1.24E-14 | 1.56E-12 |
|  | sll0271 | SGL_RS11835 | nusB | transcription antitermination factor NusB | 1.06103753 | 0.0000275 | 0.00054546 |
|  | sll0789 | SGL_RS16110 | OmpR subfamily | response regulator transcription factor | 1.632644831 | 0.00000105 | 0.0000355 |
|  | ssl0564 | SGL_RS12615 | SGL_RS12615 | helix-turn-helix transcriptional regulator | 1.003936844 | 0.003889093 | 0.027648225 |
| Intracellular trafficking secretion and vesicular transport | sll0858 | SGL_RS08155 | SGL_RS08155 | Spy/CpxP family protein refolding chaperone | 2.615378647 | 1.44E-19 | 5.87E-17 |
|  | sll1695 | SGL_RS07940 | hofG | type II secretion system protein | 1.203142572 | 0.0000746 | 0.001239739 |
| Cell wall/membrane/envelope biogenesis | sll1723 | SGL_RS06330 | SGL_RS06330 | glycosyltransferase family 4 protein | 3.993216873 | 5.77E-24 | 4.7E-21 |
|  | sll1724 | SGL_RS06325 | icsA | glycosyltransferase family 4 protein | 2.927924749 | 5.22E-21 | 2.83E-18 |
|  | sll0409 | SGL_RS13720 | menC | o-succinylbenzoate synthase | 1.054148026 | 0.006247895 | 0.039272484 |
|  | slr5056 | SGL_RS00255 | SGL_RS00255 | glycosyltransferase family 2 protein | 1.981452547 | 0.0000108 | 0.000250388 |
|  | slr5055 | SGL_RS00250 | SGL_RS00250 | WecB/TagA/CpsF family glycosyltransferase | 1.625898714 | 0.00000251 | 0.0000742 |
|  | sll0141 | SGL_RS12205 | SGL_RS12205 | efflux RND transporter periplasmic adaptor subunit | 1.832450835 | 0.000124823 | 0.001917094 |
|  | sll1835 | SGL_RS04760 | SGL_RS04760 | CsgG/HfaB family protein | 1.50313988 | 5.72E-08 | 0.00000291 |
| Replication/recombination and repair | sll0270 | SGL_RS11845 | priA | primosomal protein N' | -1.126947929 | 0.000185406 | 0.002557974 |
|  | slr7097 | SGL_RS01085 | SGL_RS01085 | alpha-ketoglutarate-dependent dioxygenase AlkB | 1.360356062 | 0.001458146 | 0.012831689 |
|  | sll1854 | SGL_RS12355 | xth | exodeoxyribonuclease III | 1.49082044 | 0.005022914 | 0.032840578 |
|  |  | SGL_RS18790 | SGL_RS18790 | IS1 family transposase | 1.477454668 | 0.00000563 | 0.000147885 |
| General function prediction only | sll0496 | SGL_RS16770 | SGL_RS16770 | LptF/LptG family permease | 1.92697079 | 0.001090126 | 0.010378513 |
|  | sll1722 | SGL_RS06335 | SGL_RS06335 | glycosyltransferase family protein | 3.501471043 | 2.94E-17 | 6.84E-15 |
|  | slr8038 | SGL_RS01315 | SGL_RS01315 | WD40 repeat domain-containing protein | -1.067120855 | 0.0000379 | 0.000709722 |
|  | sll0209 | SGL_RS13550 | SGL_RS13550 | long-chain acyl -[acyl -carrier-protein] reductase | 1.270479887 | 0.000294412 | 0.003658793 |
|  | sll0553 | SGL_RS14940 | SGL_RS14940 | alpha/beta hydrolase | 1.236351313 | 8.15E-09 | 0.000000531 |
|  | sll1491 | SGL_RS17455 | SGL_RS17455 | WD40 repeat domain-containing protein | 1.311129256 | 7.76E-10 | 5.74E-08 |
|  | sll0409 | SGL_RS13720 | menC | o-succinylbenzoate synthase | 1.054148026 | 0.006247895 | 0.039272484 |
|  | slr5054 | SGL_RS00245 | SGL_RS00245 | glycosyltransferase | 2.489564081 | 0.0000022 | 0.0000675 |
|  | slr7095 | SGL_RS01075 | SGL_RS01075 | AAA family ATPase | 1.004962559 | 0.007972848 | 0.046356415 |
|  | sll0572 | SGL_RS18260 | SGL_RS18260 | cyclic 2,3-diphosphoglycerate synthase | -2.040638521 | 0.0000082 | 0.000208609 |
|  | sll0330 | SGL_RS12965 | fabG | SDR family NAD(P)-dependent oxidoreductase | 2.150532568 | 0.0000738 | 0.001238821 |
| Function unknown | sll0185 | SGL_RS14600 | SGL_RS14600 | DUF4912 domain-containing protein | -1.144732454 | 0.000013 | 0.000296957 |
|  | sll1938 | SGL_RS04610 | SGL_RS04610 | Uma2 family endonuclease | 1.114389146 | 0.004312009 | 0.029706853 |
|  | sll0254 | SGL_RS08895 | SGL_RS08895 | FAD-dependent oxidoreductase | -1.64374593 | 0.000228131 | 0.003044235 |
|  | sll0727 | SGL_RS17735 | SGL_RS17735 | hypothetical protein | -1.172550471 | 0.0000462 | 0.000835993 |
|  | sll1464 | SGL_RS06510 | SGL_RS06510 | YdiU family protein | 1.564965179 | 0.000228013 | 0.003044235 |
|  | sll1837 | SGL_RS04740 | SGL_RS04740 | DUF1036 domain-containing protein | -1.593175536 | 4.62E-15 | 6.84E-13 |
|  | sll0788 | SGL_RS16115 | SGL_RS16115 | DUF305 domain-containing protein | 2.396015063 | 0.006072652 | 0.038391063 |
|  | sll5132 | SGL_RS00005 | SGL_RS00005 | DUF262 domain-containing protein | 1.4556421 | 0.00000886 | 0.00021519 |
|  | sll0525 | SGL_RS16965 | SGL_RS16965 | type II toxin-antitoxin system VapC family toxin | 1.649323399 | 0.000061 | 0.001055974 |
|  | sll0471 | SGL_RS15545 | SGL_RS15545 | FAD-dependent oxidoreductase | 2.945582188 | 3.44E-25 | 5.59E-22 |
|  | sll0314 | SGL_RS13285 | SGL_RS13285 | hypothetical protein | 2.629695581 | 7.94E-15 | 1.08E-12 |
|  | sll0857 | SGL_RS08160 | SGL_RS08160 | hypothetical protein | 2.146014329 | 4.38E-12 | 3.97E-10 |
|  | sll0319 | SGL_RS13245 | SGL_RS13245 | DUF3747 domain-containing protein | 1.195401937 | 2.05E-10 | 1.76E-08 |
|  | sll1586 | SGL_RS08890 | SGL_RS08890 | translocation/assembly module TamB domain-containing protein | -1.596209649 | 4.32E-10 | 3.35E-08 |
|  | sll0702 | SGL_RS02395 | SGL_RS02395 | hypothetical protein | 2.060571539 | 1.08E-08 | 6.76E-07 |
|  | sll1891 | SGL_RS10265 | SGL_RS10265 | DUF928 domain-containing protein | 1.500942588 | 2.64E-08 | 1.59E-06 |
|  | sll1738 | SGL_RS06250 | SGL_RS06250 | hypothetical protein | 1.783833576 | 4.39E-08 | 2.51E-06 |
|  | sll1696 | SGL_RS07935 | SGL_RS07935 | hypothetical protein | 2.260111471 | 4.78E-08 | 2.51E-06 |
|  |  | SGL_RS19605 | SGL_RS19605 | hypothetical protein | 1.910426729 | 7.82E-08 | 3.64E-06 |
|  | slr5053 | SGL_RS00240 | SGL_RS00240 | cyanoexosortase A system-associated protein | 2.315984535 | 9.87E-08 | 4.34E-06 |
|  | sll1570 | SGL_RS05315 | SGL_RS05315 | hypothetical protein | -1.026670346 | 7.12E-07 | 2.58E-05 |
|  | sll1267 | SGL_RS07120 | SGL_RS07120 | hypothetical protein | 1.109180023 | 7.65E-07 | 2.71E-05 |
|  |  | SGL_RS19390 | SGL_RS19390 | hypothetical protein | 1.562799835 | 2.29E-06 | 6.91E-05 |
|  | sll1265 | SGL_RS07125 | SGL_RS07125 | translocation/assembly module TamB domain-containing protein | -1.239938336 | 2.83E-06 | 8.24E-05 |
|  | sll0470 | SGL_RS15550 | SGL_RS15550 | DUF2808 domain-containing protein | 1.343296715 | 3.95E-06 | 0.000110749 |
|  | sll1507 | SGL_RS04105 | SGL_RS04105 | alpha/beta hydrolase | 1.174991912 | 5.63E-06 | 0.000147885 |
|  | sll1929 | SGL_RS04705 | comEc | ComEC/Rec2 family competence protein | -1.149856951 | 8.45E-06 | 0.000211628 |
|  | sll1667 | SGL_RS03105 | mom72 | tetratricopeptide repeat protein | 1.609757929 | 1.51E-05 | 0.000333213 |
|  | sll1089 | SGL_RS11055 | SGL_RS11055 | hypothetical protein | -1.017736735 | 1.73E-05 | 0.000371867 |
|  | sll0350 | SGL_RS12875 | SGL_RS12875 | DUF3769 domain-containing protein | 1.392442039 | 1.79E-05 | 0.000374532 |
|  |  | SGL_RS14355 | SGL_RS14355 | hypothetical protein | -1.035237318 | 2.02E-05 | 0.000416054 |
|  | sll1426 | SGL_RS09265 | SGL_RS09265 | hypothetical protein | 1.022133026 | 2.84E-05 | 0.000557688 |
|  |  | SGL_RS20205 | ggpR | glucosylglycerol biosynthesis transcriptional repressor GgpR | -1.598776076 | 6.88E-05 | 0.001167342 |
|  | ssl0467 | SGL_RS08910 | SGL_RS08910 | hypothetical protein | 1.366986167 | 0.00013231 | 0.00199445 |
|  | sll1726 | SGL_RS06315 | SGL_RS06315 | phosphotransferase | 1.988883008 | 0.000148238 | 0.00213568 |
|  |  | SGL_RS19440 | SGL_RS19440 | hypothetical protein | 2.105980014 | 0.000146938 | 0.00213568 |
|  | slr5051 | SGL_RS00230 | SGL_RS00230 | hypothetical protein | 1.109439554 | 0.000161055 | 0.002279977 |
|  | sll0853 | SGL_RS08180 | SGL_RS08180 | phycobiliprotein lyase | 1.354803031 | 0.000165915 | 0.002328536 |
|  | sll0623 | SGL_RS17335 | SGL_RS17335 | hypothetical protein | 1.191864413 | 0.000231055 | 0.003058198 |
|  | sll0930 | SGL_RS03460 | SGL_RS03460 | hypothetical protein | 1.191263848 | 0.000269418 | 0.003426658 |
|  | sll0558 | SGL_RS01890 | SGL_RS01890 | GUN4 domain-containing protein | 1.133483005 | 0.000345987 | 0.004172348 |
|  | sll1737 | SGL_RS06255 | SGL_RS06255 | Tic20 family protein | 1.22297608 | 0.000356726 | 0.004256611 |
|  | sll0931 | SGL_RS03455 | SGL_RS03455 | tetratricopeptide repeat protein | 1.537588045 | 0.000393857 | 0.004515486 |
|  | sll1527 | SGL_RS11605 | SGL_RS11605 | glycosyltransferase | -1.051030473 | 0.000417072 | 0.00471523 |
|  | ssl1972 | SGL_RS03760 | SGL_RS03760 | hypothetical protein | 1.110438634 | 0.000420845 | 0.004725071 |
|  | sll0819 | SGL_RS09810 | psaF | photosystem I reaction center subunit III | -1.112901129 | 0.00060881 | 0.006313006 |
|  | ssl0453 | SGL_RS08995 | nblA | NblA/ycf18 family protein | 1.170183327 | 0.000657066 | 0.006770277 |
|  | sll1471 | SGL_RS17600 | cpcG | phycobilisome rod-core linker polypeptide | 1.260901532 | 0.000734388 | 0.007380143 |
|  | sll1239 | SGL_RS09630 | SGL_RS09630 | hypothetical protein | 1.902112179 | 0.000884956 | 0.008731565 |
|  | sll0253 | SGL_RS08900 | SGL_RS08900 | DUF6335 family protein | -1.18766402 | 0.001125365 | 0.010651708 |
|  | slr7094 | SGL_RS01070 | SGL_RS01070 | hypothetical protein | 1.033238201 | 0.001258196 | 0.011507548 |
|  | ssl0452 | SGL_RS09000 | nblA | NblA/ycf18 family protein | 1.211827431 | 0.001291031 | 0.011741894 |
|  | sll1107 | SGL_RS18180 | SGL_RS18180 | ABC transporter permease | 1.681689296 | 0.001373763 | 0.012221232 |
|  | sll1796 | SGL_RS05875 | petJ | c-type cytochrome | -1.246979482 | 0.001614457 | 0.013906538 |
|  | sml0008 | SGL_RS09805 | psaJ | photosystem I reaction center subunit IX | -1.00176575 | 0.001656882 | 0.014122537 |
|  | ssr5121 | SGL_RS00570 | SGL_RS00570 | hypothetical protein | 2.280822892 | 0.002080268 | 0.017104428 |
|  | sll0860 | SGL_RS07310 | SGL_RS07310 | DUF3110 domain-containing protein | 1.388021058 | 0.002303262 | 0.018380936 |
|  |  | SGL_RS20110 | SGL_RS20110 | hypothetical protein | -1.026882859 | 0.002574253 | 0.020052072 |
|  | sll0742 | SGL_RS16415 | SGL_RS16415 | DUF29 domain-containing protein | 1.563765245 | 0.002688847 | 0.020844965 |
|  | sll0376 | SGL_RS14490 | SGL_RS14490 | hypothetical protein | -1.386075374 | 0.003001432 | 0.022833326 |
|  | sll1245 | SGL_RS10145 | cytM | cytochrome c | 1.306063787 | 0.003388666 | 0.025076129 |
|  | slr7098 | SGL_RS01090 | SGL_RS01090 | hypothetical protein | 1.23665899 | 0.003747475 | 0.026995083 |
|  | sll1398 | SGL_RS02070 | psb28 | photosystem II reaction center protein Psb28 | 1.060913859 | 0.003950521 | 0.027841764 |
|  | slr5127 | SGL_RS00600 | SGL_RS00600 | hypothetical protein | 1.545709281 | 0.003997965 | 0.028054689 |
|  | sml0007 | SGL_RS07800 | ycf32 | photosystem II protein Y | 1.594025089 | 0.004324646 | 0.029706853 |
|  |  | SGL_RS13295 | rrf | 5S ribosomal RNA | 1.318136329 | 0.004270821 | 0.029706853 |
|  | sll1333 | SGL_RS17110 | SGL_RS17110 | hypothetical protein | -1.326810316 | 0.005318479 | 0.034633935 |
|  | sll1757 | SGL_RS07675 | SGL_RS07675 | HpsJ family protein | 1.12461679 | 0.005855959 | 0.037533472 |
|  | sll0710 | SGL_RS02320 | SGL_RS02320 | hypothetical protein | 1.514573173 | 0.007076703 | 0.042512443 |
|  | slr7037 | SGL_RS00795 | SGL_RS00795 | DUF3854 domain-containing protein | -1.877577897 | 0.008057457 | 0.046516097 |
|  | slr5073 | SGL_RS00330 | SGL_RS00330 | hypothetical protein | 1.136819445 | 0.008555962 | 0.048703168 |
|  | sll0567 | SGL_RS18290 | fur | transcriptional repressor | 0.776772837 | 0.025391691 | 0.106540395 |
|  | sll1291 | SGL_RS05095 | PatA | response regulator | -0.138725376 | 0.034380913 | 0.130931152 |
|  | sll1292 | SGL_RS05090 | CheY | response regulator | -0.411472143 | 0.005959077 | 0.038044619 |
|  | sll1293 | SGL_RS05085 | SGL_RS05085 | chemotaxis protein CheW | -0.453209195 | 0.003685447 | 0.026785299 |
|  | sll1371 | SGL_RS10605 | SGL_RS10605 | Crp/Fnr family transcriptional regulator | -0.431528435 | 0.019032483 | 0.086792388 |

**Table S2.** DEGs identified between the Δ*zur* mutant and the WT by RNA-seq in *Synechocystis* sp. PCC 6803.
